# Confirmed effects of candidate variants for milk production, udder health, and udder morphology in dairy cattle

**DOI:** 10.1186/s12711-020-00575-1

**Published:** 2020-10-01

**Authors:** Thierry Tribout, Pascal Croiseau, Rachel Lefebvre, Anne Barbat, Mekki Boussaha, Sébastien Fritz, Didier Boichard, Chris Hoze, Marie-Pierre Sanchez

**Affiliations:** 1 Université Paris-Saclay, INRAE, AgroParisTech, GABI, 78350 Jouy-en-Josas, France; 2 Allice, 75012 Paris, France

## Abstract

**Background:**

Over the last years, genome-wide association studies (GWAS) based on imputed whole-genome sequences (WGS) have been used to detect quantitative trait loci (QTL) and highlight candidate genes for important traits. However, in general this approach does not allow to validate the effects of candidate mutations or determine if they are truly causative for the trait(s) in question. To address these questions, we applied a two-step, within-breed GWAS approach on 15 traits (5 linked with milk production, 2 with udder health, and 8 with udder morphology) in Montbéliarde (MON), Normande (NOR), and Holstein (HOL) cattle. We detected the most-promising candidate variants (CV) using imputed WGS of 2515 MON, 2203 NOR, and 6321 HOL bulls, and validated their effects in three younger populations of 23,926 MON, 9400 NOR, and 51,977 HOL cows.

**Results:**

Bull sequence-based GWAS detected 84 QTL: 13, 10, and 30 for milk production traits; 3, 0, and 2 for somatic cell score (SCS); and 8, 2 and 16 for udder morphology traits, in MON, NOR, and HOL respectively. Five genomic regions with effects on milk production traits were shared among the three breeds whereas six (2 for production and 4 for udder morphology and health traits) had effects in two breeds. In 80 of these QTL, 855 CV were highlighted based on the significance of their effects and functional annotation. The subsequent GWAS on MON, NOR, and HOL cows validated 8, 9, and 23 QTL for production traits; 0, 0, and 1 for SCS; and 4, 1, and 8 for udder morphology traits, respectively. In 47 of the 54 confirmed QTL, the CV identified in bulls had more significant effects than single nucleotide polymorphisms (SNPs) from the standard 50K chip. The best CV for each validated QTL was located in a gene that was functionally related to production (36 QTL) or udder (9 QTL) traits.

**Conclusions:**

Using this two-step GWAS approach, we identified and validated 54 QTL that included CV mostly located within functional candidate genes and explained up to 6.3% (udder traits) and 37% (production traits) of the genetic variance of economically important dairy traits. These CV are now included in the chip used to evaluate French dairy cattle and can be integrated into routine genomic evaluation.

## Background

The increasing amount of whole-genome sequence (WGS) data for bovine species [1,2], combined with the regular use of high-throughput genotyping for genomic selection in cattle, has made it possible to run genome-wide association studies (GWAS) directly on imputed sequence data in large cohorts of animals for complex traits of economic importance. Since the first GWAS on imputed WGS in dairy cattle published 6 years ago [2,3], several sequence-based GWAS have been conducted in dairy or beef cattle. However, in their review of the applications and outcomes of the “1000 Bull Genomes” project, Hayes and Daetwyler [4] noted that, even if the majority of polymorphisms within a cattle population can be tested using readily available whole-genome sequence data, the unambiguous identification of an individual mutation as causative for a complex trait remains the exception rather than the norm. GWAS on imputed WGS enables the targeting of small genomic regions such as genes, but the identification of causal polymorphisms is much less straightforward. Difficulties in pinpointing causal mutations arise from (i) the long-range linkage disequilibrium (LD) that exists in cattle breeds, which usually results in the detection of a set of variants in high LD rather than a single causal variant, (ii) variability in imputation accuracy, which may favor a variant in LD with the causal mutation rather than the mutation itself, and (iii) poor annotation of the bovine genome, in particular in regulatory regions, which makes it difficult to distinguish the best functional candidate in a set of variants.

Beyond providing a better understanding of the underlying biology of complex traits, the identification of causal mutations could be beneficial for genomic evaluation, especially across populations. The integration of causal mutations into genomic evaluation models could increase the accuracy of predictions and ensure the persistence of these models across generations or for distantly related individuals [5]. Models that have been developed in major breeds might then be more easily transposed to smaller breeds, for which accurate genomic evaluation is difficult to implement. In addition, models with causal variants can account for interactions between genes more easily. However, to avoid the integration of false-positive candidate variants into models, their effects must first be validated in other populations that are as independent as possible. The Eurogenomics custom single nucleotide polymorphism (SNP) chip, which has been developed for bovine genomic selection, appears to be an ideal tool for this purpose. It contains an add-on feature that can be updated once or twice a year, and it is widely used in multiple breeds [6], which makes it possible to validate the effects of candidate variants detected by GWAS in different large populations.

In dairy cattle in particular, production traits are of major importance. First and foremost, high milk production is conditioned by a good and healthy udder. Mastitis is the most important health problem in dairy cattle and has an unfavorable genetic correlation with milk yield [7,8]. Udder morphology is closely linked to sustainable milk production and is also associated with mastitis resistance [8] and longevity [9]. Thus, there are great benefits to considering all of these traits in the same study.

In order to disentangle the biological relationship between these complex traits and propose candidate causative variants, the objectives of this study were to identify genes, and the polymorphisms within them, that are responsible for the genetic variation in traits related to milk production, udder health, and udder morphology in the three main French dairy cattle breeds: Holstein (HOL), Montbéliarde (MON), and Normande (NOR). First, we conducted within-breed GWAS using imputed WGS of bulls with performances (Part I); then, we validated the effects of the candidate causal variants highlighted in the initial detection by performing within-breed GWAS in statistically independent populations of cows (Part II).

## Methods

This study comprised two parts. Part I consisted of identifying QTL and candidate variants from sequence-based GWAS of three bull populations. Part II aimed at confirming their effects by conducting a GWAS using the candidate variants from Part I and SNPs from the 50K SNP chip in three cow populations. For this study, we did not perform any experiments on animals; thus, no ethical approval was required.

### Part I: Identification of candidate causative variants in bulls

#### Animals, phenotypes, and genotypes

To identify QTL and candidate variants, GWAS were performed at the sequence level on populations of bulls from the three main French dairy cattle breeds, i.e. HOL, MON, and NOR, for which genotypes and data on daughters’ performance are available until 2014.

Bulls were genotyped with the Illumina Bovine SNP50 BeadChip (50K; Illumina Inc., San Diego, CA). Most key ancestors were genotyped at the high-density (HD) level (777k SNP, Illumina Bovine HD Beadchip; Illumina Inc., San Diego, CA) and the genome of some of them was sequenced (WGS), as shown in Table 1. We applied the following quality control filters to the 50K and HD genotypes: an individual call rate higher than 0.95, a SNP call rate higher than 0.90, a minor allele frequency (MAF) higher than 0.01 in at least one breed, and genotype frequencies had to be in Hardy–Weinberg equilibrium with P > 10^−4^.

**Table 1:** Number of bulls with 50k SNP (50K), 777k SNP (HD), or whole-genome sequence (WGS) genotypes in each breed

Breed	50K	HD	WGS	Total
Holstein	6321	776	288	6321
Montbéliarde	2515	522	28	2515
Normande	2203	546	24	2203

In total, we analyzed 16 (HOL and MON) or 15 (NOR) routinely collected traits:

- Five milk production traits: milk yield (MY), protein yield (PY), fat yield (FY), protein content (PC), and fat content (FC);
- Two udder health traits: somatic cell score (SCS) and clinical mastitis (CM). SCS was defined as SCS = 3 + log_2_(SCC/100,000) and averaged over monthly measures within lactation, with SCC being the number of somatic cells per ml of milk. CM was defined within lactation as a 0/1 trait with 1 corresponding to the occurrence of at least one clinical case before 150 days in milk;
- Eight udder morphology traits, recorded by a type classifier during a classification visit: udder support (US), udder depth (UD), fore udder attachment (FUA), rear udder height (RUH), fore teat distance (FTD), udder balance (UB), and teat orientation (TO) in all breeds, teat length (TL) in MON and HOL. Scores, ranging from 1 to 9, were recorded only once per cow in first lactation;
- Milking speed score (MSS), a subjective appraisal ranging from 1 to 5, given by the farmer and recorded with morphology traits.

In this paper, for convenience, health traits, type traits and milking speed are referred to as udder traits.

For all traits, the phenotypes used in the analyses were the daughter yield deviations (DYD) of each bull, defined as the average value of daughters’ performance, adjusted for fixed and non-genetic random effects and for the breeding value of their dams [10]. DYD are produced by the French national genetic evaluation systems of HOL, MON, and NOR populations with the models described at https://interbull.org/ib/geforms [11]. Mean reliabilities for all traits, excluding CM, ranged from 0.74 to 0.94, depending on the breed and on the trait (Table 2). Mean reliabilities for CM were lower (0.40 for NOR, 0.43 for MON and HOL), which was a result of both the lower heritability (about 0.02) of this trait and the fact that it began being recorded on farms more recently than other traits.

**Table 2:** Number of bulls with genotypes and phenotypes (DYD) and average reliability of their phenotypes for each trait, in Montbéliarde (MON), Normande (NOR), and Holstein (HOL) cattle

Type of trait	Trait and abbreviation	Number of bulls with DYD			Reliability of DYD mean (*sd*)		
		MON	NOR	HOL	MON	NOR	HOL
Milk production	Milk yield (kg) MY	2434	2175	6262	0.91 *(0.09)*	0.89 *(0.11)*	0.92 *(0.05)*
	Fat content (%) FC	2434	2175	6262	0.93 *(0.08)*	0.92 *(0.10)*	0.94 *(0.04)*
	Protein content (%) PC	2434	2175	6262	0.93 *(0.08)*	0.92 *(0.10)*	0.94 *(0.04)*
	Fat yield (kg) FY	2434	2175	6262	0.91 *(0.09)*	0.89 *(0.11)*	0.92 *(0.05)*
	Protein yield (kg) PY	2434	2175	6262	0.91 *(0.09)*	0.89 *(0.11)*	0.92 *(0.05)*
Udder health	Clinical mastitis CM	1857	1427	4959	0.43 *(0.21)*	0.40 *(0.22)*	0.43 *(0.21)*
	Somatic cell score SCS	2438	2203	6318	0.87 *(0.07)*	0.85 *(0.07)*	0.88 *(0.06)*
Udder morphology	Udder support US	2494	2180	6311	0.83 *(0.07)*	0.87 *(0.06)*	0.82 *(0.08)*
	Udder depth UD	2511	2020	6319	0.90 *(0.05)*	0.83 *(0.07)*	0.88 *(0.06)*
	Fore udder attachment FUA	2500	2164	5959	0.86 *(0.07)*	0.82 *(0.07)*	0.83 *(0.08)*
	Rear udder height RUH	2498	2147	6107	0.85 *(0.07)*	0.74 *(0.10)*	0.80 *(0.09)*
	Teat length TL	2515	–	6321	0.92 *(0.05)*	–	0.89 *(0.05)*
	Fore teat distance FTD	2509	2032	6319	0.89 *(0.06)*	0.86 *(0.07)*	0.88 *(0.06)*
	Udder balance UB	2478	2164	6275	0.77 *(0.09)*	0.81 *(0.08)*	0.81 *(0.09)*
	Teat orientation TO	2500	2175	6318	0.86 *(0.06)*	0.85 *(0.06)*	0.85 *(0.07)*
Milking ease	Milking speed score MSS	2500	2164	6300	0.86 *(0.07)*	0.80 *(0.08)*	0.79 *(0.09)*

#### Imputation to whole-genome sequences

Using the UMD3.1 assembly, genotypes of all bulls were imputed to WGS with the FImpute software, which accurately and quickly processes large datasets [12]. A two-step process was performed in order to improve imputation accuracy: from 50K to HD, and then from HD to WGS [13]. All imputations were performed separately for each breed using either a breed-specific (from 50K to HD SNPs) or a multi-breed (from HD SNPs to WGS) reference panel depending on the targeted density [14]. In each breed, imputations to the HD SNP level were performed using a within-breed reference population that included, respectively, 522 MON, 546 NOR, and 776 HOL bulls that had been genotyped with the Illumina BovineHD BeadChip (Illumina Inc., San Diego, CA). WGS variants were imputed from HD SNP genotypes using WGS variants of the 1147 *Bos taurus* bulls from Run4 of the 1000 Bull Genomes Project [1]; these bulls represented 27 cattle breeds, and included 288 HOL, 28 MON, and 24 NOR individuals. WGS variants were selected by applying the protocol defined by the 1000 Bull Genomes consortium [1,2]. First, short reads were filtered for quality and aligned to the UMD3.1 reference sequence [15], and small genomic variations (SNPs and InDels) were detected using SAMtools 0.0.18 [16]. Raw variants were then filtered as described in Boussaha et al. [15] to produce a dataset of 26,738,438 autosomal variants. Finally, filtered variants were annotated using the Ensembl variant effect predictor pipeline v81 [17], and the effects of amino-acid changes were predicted using the SIFT tool [18]. Imputation accuracies were estimated in the MON and HOL datasets by calculating genotypic concordance rates; these values reached 0.90 and 0.94, respectively [19]. Although the number of sequenced bulls was slightly lower in NOR than in MON, they contributed a higher proportion of the genes of the population and we assumed that imputation accuracy was similar in both breeds. Only variants with a MAF higher than 0.1% were retained for within-breed association analyses, i.e. around 12 million variants in each breed.

#### Whole-genome sequence association analyses

We performed within-breed and single-trait association analyses between all 12 million polymorphic variants (MAF ≥ 0.001) and the traits described in Table 2. All association analyses were performed using the *mlma* option of GCTA software (version 1.24), which applies a mixed linear model that includes the variant to be tested [20]:

1 $${\mathbf{y}} = {\mathbf{1}}\mu + {\mathbf{x}}b + {\mathbf{u}} + {\mathbf{e}}$$

where ${\mathbf{y}}$ is the vector of DYD standardized by the genetic standard deviation of the trait in the considered breed ($\sigma_{u\_pop}$); $\mu$ is the overall mean; $b$ is the additive fixed effect of the variant to be tested for association; ${\mathbf{x}}$ is the vector of imputed genotypes, coded 0, 1, or 2 (number of copies of the second allele); ${\mathbf{u}} \sim N\left( {0, {\mathbf{G\sigma }}_{\text{u}}^{2} } \right)$ is the vector of random polygenic effects, with ${\mathbf{G}}$ the genomic relationship matrix (GRM) calculated using the HD SNP genotypes, and $\sigma_{u}^{2}$ the polygenic variance, estimated based on the null model $\left( {{\mathbf{y}} = {\mathbf{1}}\mu + {\mathbf{u}} + {\mathbf{e}}} \right)$ and then fixed while testing for the association between each variant and the trait of interest; and ${\mathbf{e}} \sim N\left( {0, {\mathbf{I}}\varvec{\sigma}_{\text{e}}^{2} } \right)$ is the vector of random residual effects, with ${\mathbf{I}}$ the identity matrix and $\sigma_{\text{e}}^{2}$ the residual variance. Because the variability of DYD reliability was limited, residuals were assumed to have a homogeneous variance.

In order to account for multiple testing, the Bonferroni correction was applied to the thresholds by considering 8 million independent tests, after pruning for complete linkage disequilibrium. Therefore, the 5% genome-wide threshold of significance corresponded to a nominal *P*-value of 6.3 $\times$ 10^−9^ (− log_10_(*P*) = 8.2). When a given trait was significantly affected by multiple variants, the variants that were located less than 1 million base-pairs (Mbp) apart were grouped together. The bounds of the confidence intervals (CI) of each region were then determined by considering the positions of variants that were included in the upper third of the peak (individual CI). For a given trait in a given breed, CI that overlapped or were less than 1 Mbp away from each other were grouped in a QTL region. For each QTL, we then defined two CI: (1) a TOP-CI determined by the bounds of the individual CI in which we found the most significant results in the region and (2) an EXT-CI with bounds determined by the outermost positions after all overlapping individual CI were grouped. When only a single individual CI was present in a given region, TOP-CI and EXT-CI were identical. For each trait, the proportion of genetic variance explained by each QTL was estimated by $\sigma_{{{\text{g}}\_QTL}}^{2} = 2 p_{ms} \left( {1 - p_{ms} } \right) \hat{b}_{ms}^{2}$, with $p_{ms}$ and $\hat{b}_{ms}$ the frequency and the estimated allelic substitution effect in genetic standard deviation units, respectively, of the variant with the most significant effect ($ms$) in the QTL region.

### Selection of candidate variants from sequence-based GWAS results

**Fig. 1:**
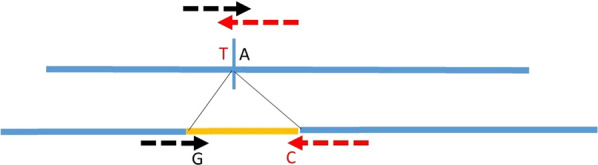
Design of the molecular test for a structural variant in a SNP chip (example of an insertion). Using the black arrow as a primer, the *G* allele reveals the insertion and the *A* allele the absence of insertion. A confirmation can be obtained with a second test on the other side of the insertion (primers = red arrows). Allele *C* reveals the insertion and T the absence of insertion

Within each of the QTL regions detected in the sequence-based GWAS, we selected the most plausible variants (SNPs or small InDels) to explain the effects we observed. About 900 variants could be added on the custom part of the chip. Variant selection was performed within breed, trait and individual QTL. A similar number of variants was a priori allocated to each individual QTL. Consequently, due to the number of QTL finally detected, about 10 variants were selected for each individual QTL. Candidate variants with a MAF higher than 0.02 were chosen based first on the level of significance of their effect. For top variants with similar significance levels, the best candidates were discriminated based on their functional annotation with a priority for genic variants in coding (missense and loss of function) and regulatory regions. The selected variants, 855 in total, were then included on the custom part of version 6 of the Illumina EuroG10K BeadChip [6]. When these variants were InDels, their breakpoints were tested as done for SNPs, as described in Fig. 1 [6].

### Part II: Validation of the effects of candidate causative variant in cows

The second part of this study was dedicated to validating the effects of these QTL regions and the candidate variants identified within them. To this end, we tested the effects of the candidate variants, as well as those of the 50K SNPs, on the performance of three statistically independent datasets of HOL, MON, and NOR cows.

#### Genotyping and imputations

Of all the cows genotyped for the purpose of genomic selection in France, we found 51,977 HOL, 23,926 MON, and 9400 NOR cows, born from 2014, whose production and udder phenotypes were not included in the DYD calculations of bulls used in Part I. Thus, phenotypes of bulls used in Part I and cows used in Part II were statistically independent. These cows were genotyped using the BovineSNP50 BeadChip (Illumina Inc.) or the customized low-density EuroG10K BeadChip (versions 1 to 5; Illumina Inc.). Missing genotypes were imputed with the FImpute software [12] in a two-step procedure. Generic markers from the BovineSNP50 Beadchip were imputed using all 50K genotyped animals as the reference, as per the routine procedure of the French evaluation system. Then, customized markers were imputed using as a reference all males and females (with and without phenotypes) that had been genotyped using the EuroG10K BeadChip (versions 1 to 6), i.e. 52,630 HOL, 32,373 MON, and 12,316 NOR animals. After the imputation process, all cows with phenotypes had genotypes for the variants of both the 50K Beadchip and EuroG10K BeadChip version 6, including the candidate variants detected in Part I. The accuracy of imputation was assessed by calculating mean squared correlations (R^2^) between imputed and true genotypes in a validation set of variants with MAF ≥ 1%; these values were equivalent in the three breeds and reached on average 97% for the 50K SNPs and 96% for the CV.

#### GWAS analyses

Single-trait association analyses were performed between all of the polymorphic variants of the 50K and EuroG10K Beadchips with MAF ≥ 1% (46,753, 44,832, and 44,659 SNPs in HOL, MON and NOR, respectively) and the 16 (HOL and MON) or 15 (NOR) traits described in Table 2. The phenotypes considered were the yield deviations (YD) of each cow, as estimated in the French national genetic evaluation programs of the HOL, MON, and NOR populations. YD can be interpreted as a cow’s performance, adjusted for environmental effects; for traits with repeated measures, it is the weighted mean of the cow’s performance, adjusted for non-genetic effects. As for bulls DYD, YD are by-products of the French evaluation system [11]. As in Part I, we used GCTA [20] and applied model (1) on the vector ${\mathbf{y}}$ of the YD of the cows, considering ${\mathbf{G}}$, the genomic relationship matrix (GRM), calculated with the 50K SNP genotypes. The SNP effect was considered significant if its −log_10_(*P*) value was higher than 6 (5% genome-wide threshold after Bonferroni correction, i.e. 10^−6^). As before, all variant positions were from the UMD3.1 assembly.

## Results

### Part I: Results from the bull sequence-based GWAS

**Fig. 2:**
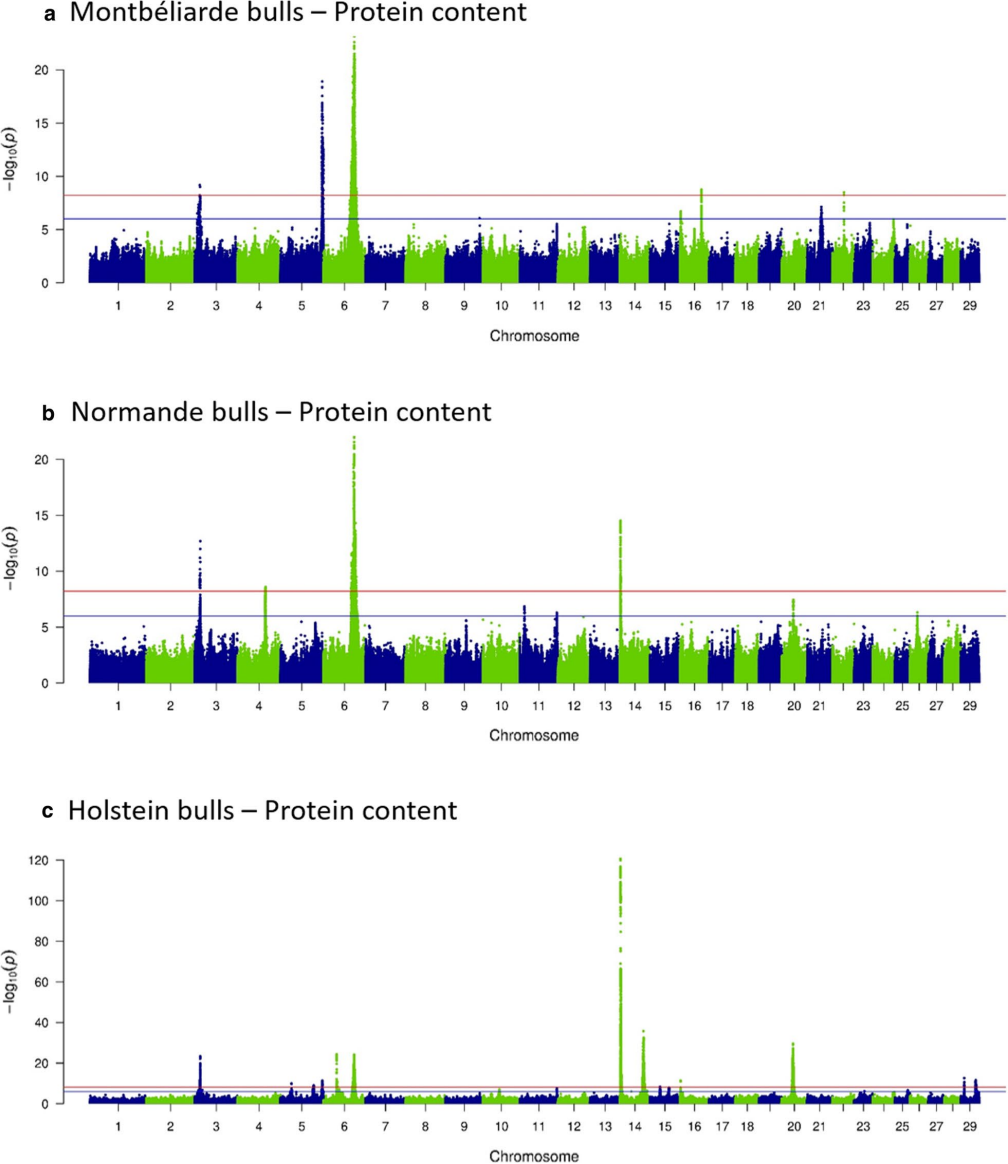
Sequence-based GWAS: – log_10_(P) plotted against the position on *Bos taurus* autosomes of variants linked with protein content in **a** Montbéliarde, **b** Normande, and **c** Holstein bulls

**Fig. 3:**
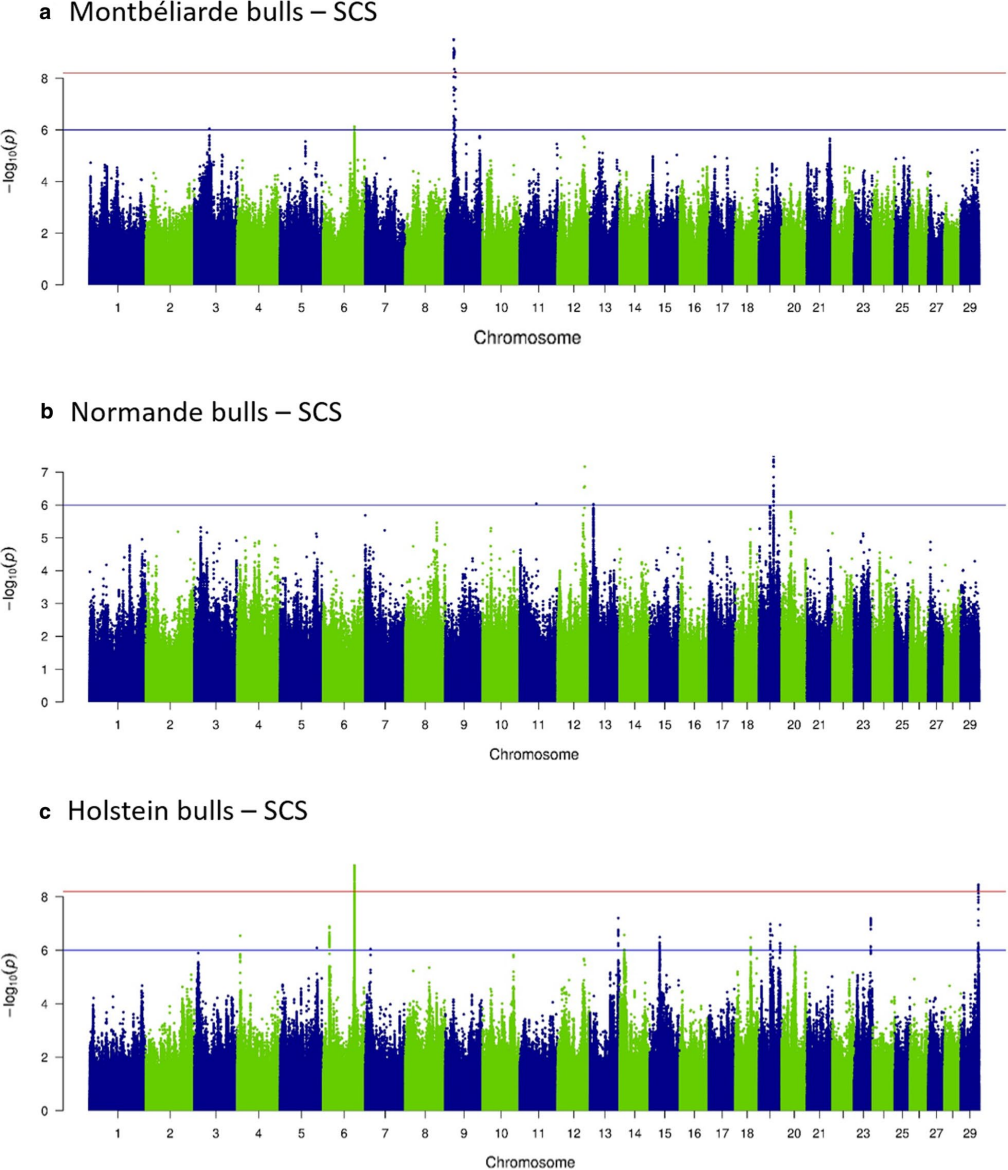
Sequence-based GWAS: – log_10_(P) plotted against the position on *Bos taurus* autosomes of variants linked with somatic cell score (SCS) in **a** Montbéliarde, **b** Normande, and **c** Holstein bulls

**Fig. 4:**
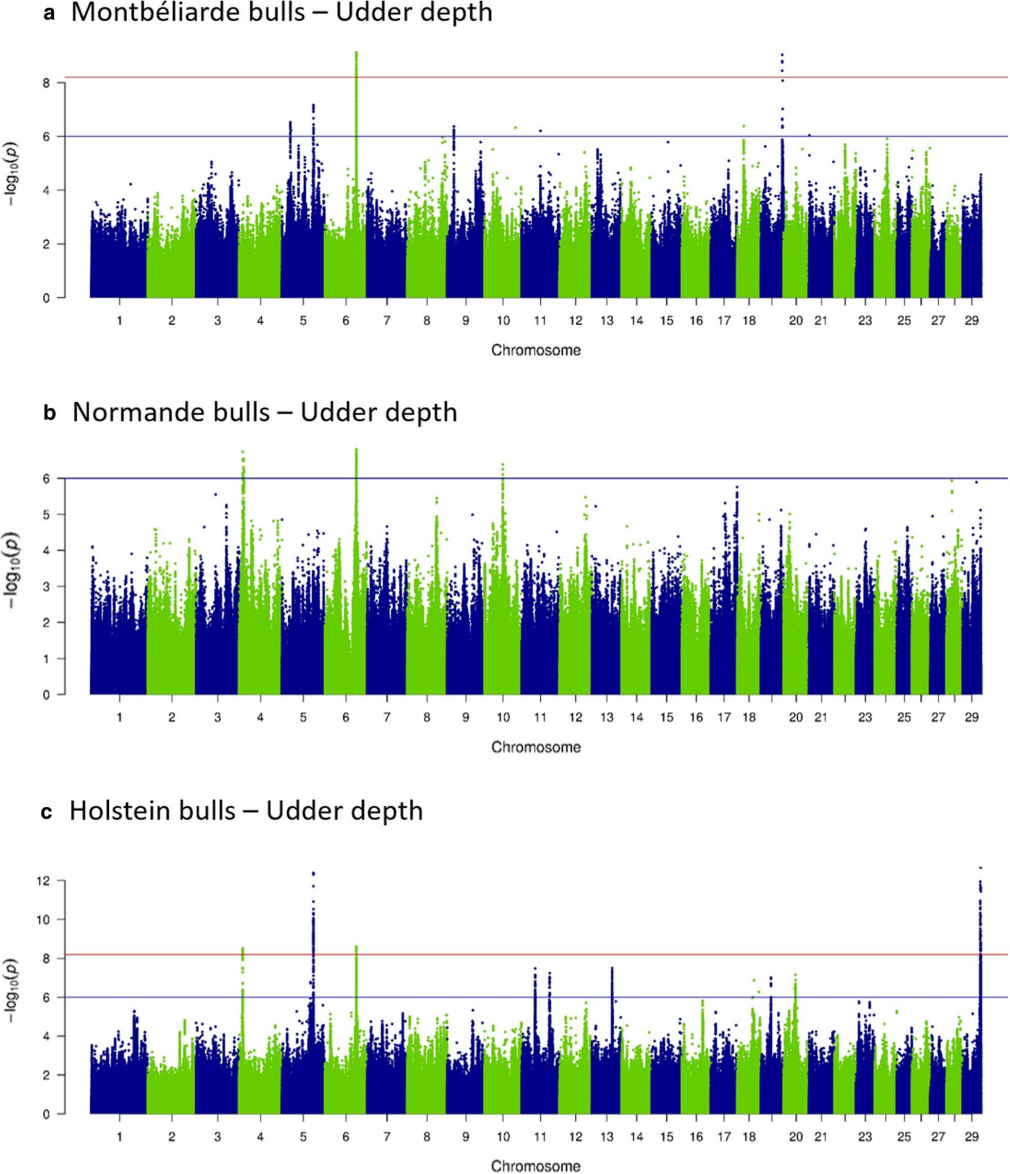
Sequence-based GWAS: –log_10_(P) plotted against the position on *Bos taurus* autosomes of variants linked with udder depth in **a** Montbéliarde, **b** Normande, and **c** Holstein bulls

GWAS of imputed whole-genome sequences of MON, NOR, and HOL bulls revealed 24, 12, and 48 QTL, respectively, with significant effects (−log_10_(*P*) ≥ 8.2; Table 3) on production (Fig. 2) or udder morphology and udder health traits (Figs. 3 and 4). At least one QTL was identified for all traits in the HOL dataset with the exception of CM, but no QTL was found for five traits in MON (PY, CM, TL, FTD, and TO) and nine traits in NOR (PY, CM, SCS, UD, FUA, FTD, UB, TO, and MSS). For the three breeds, we detected a larger number of QTL linked with milk production (13, 10, and 30 in MON, NOR and HOL, respectively), than with udder morphology (8, 2, and 16, respectively) or udder health traits (3, 0, and 2, respectively). Each QTL explained from 1.1 to 11.1% of the genetic variance of its associated trait in MON, 1.7 to 18.4% in NOR, and 0.3 to 26.8% in HOL. In each of the three breeds, the largest number of QTL was found for PC (6, 5, and 11 in MON, NOR, and HOL, respectively; Fig. 2), and their cumulative effects explained 17.2% (MON), 20.0% (NOR), and 27.7% (HOL) of the genetic variance of this trait. In each breed, the QTL that explained the largest percentage of the genetic variance of a trait was associated with FC, and the cumulative effects of all the QTL detected for this trait accounted for 19.7%, 23.2%, and 37% of the total genetic variance in MON, NOR, and HOL, respectively. In the three breeds, both the number of QTL and their individual estimated effects were lower for udder traits than for production traits; consequently, the QTL that were identified for these traits explained a smaller part of their genetic variance. In addition, in contrast to the results for production traits, the udder morphology or health trait which had the largest percentage of genetic variance explained by QTL was different among breeds: SCS with 6.3% in MON, RUH with 5.2% in NOR, and UD with 6.3% in HOL.

**Table 3:** Number of QTL and total (TOT), lowest (Min), and largest (Max) percentages of genetic variance of the trait explained by the QTL detected in sequence-based GWAS performed on bulls in each breed

Trait*	Montbéliarde				Normande				Holstein			
	#QTL	TOT	Min	Max	#QTL	TOT	Min	Max	#QTL	TOT	Min	Max
MY	1	1.6	1.6	1.6	1	2.0	2.0	2.0	3	10.0	1.1	7.8
FC	5	19.7	1.8	11.1	3	23.2	1.7	18.4	8	37.0	0.5	26.8
PC	6	17.2	1.2	7.0	5	20.0	2.0	8.0	11	27.7	0.7	8.2
FY	1	1.1	1.1	1.1	1	3.0	3.0	3.0	4	13.8	0.6	10.9
PY	0				0				4	3.7	0.3	1.7
CM	0				0				0	0	0	0
SCS	3	6.3	1.9	2.2	0				2	2.6	0.8	1.8
US	1	4.3	4.3	4.3	1	2.5	2.5	2.5	1	1.6	1.6	1.6
UD	2	4.9	1.8	3.1	0				4	6.3	0.8	1.7
FUA	1	3.9	3.9	3.9	0				2	3.1	1.5	1.6
RUH	1	3.2	3.2	3.2	1	5.2	5.2	5.2	2	3.9	1.0	2.9
TL	0				–	–	–	–	1	3.4	3.4	3.4
FTD	0				0				2	3.6	1.6	1.9
UB	1	5.1	5.1	5.1	0				1	1.2	1.2	1.2
TO	0				0				1	0.9	0.9	0.9
MSS	2	3.9	1.9	2.0	0				2	2.2	1.0	1.2

*For the description of the traits see Table 2

As described in the “Methods” section, for each QTL we defined two confidence intervals (CI) using either the CI of the most significant individual QTL (TOP-CI) or by the inclusion of all individual CI of the QTL within the region (EXT-CI). Genomic annotations of the variants located in the 84 QTL regions (TOP-CI or EXT-CI) are summarized over all traits and breeds in Table 4. Considering all QTL together, 11,696 and 20,798 distinct variants with significant effects were located within the TOP-CI and EXT-CI regions, respectively. These variants were mainly located in intergenic regions (56.8 and 59.7% for TOP-CI and EXT-CI, respectively) or in introns of genes (28.1 and 29.2%, respectively) of the bovine genome. Only 50 (0.43%, TOP-CI) and 66 (0.32%, EXT-CI) of the variants were missense. The remaining variants were located in putative regulatory regions of the bovine genome: mainly, the upstream and downstream regions and, to a lesser extent, the 3′ UTR, 5′ UTR, and splicing regions of genes.

**Table 4:** Genomic annotations of variants included within the confidence intervals (CI) of the 84 QTL, defined using either the CI of the most significant individual QTL (TOP-CI) or all the individual CI (EXT-CI) within each QTL region

Functional annotation	TOP-CI: CI of the QTL with the most significant effect		EXT-CI: Extended CI	
	Number	%	Number	%
Intergenic	6642	56.8	12,421	59.7
Upstream	764	6.5	965	4.6
Downstream	773	6.6	1039	5.0
3’ UTR	41	0.35	50	0.24
5′ UTR	14	0.12	14	0.07
Intronic	3286	28.1	6077	29.2
Synonymous	105	0.90	133	0.64
Non-coding transcript exon	4	0.03	6	0.03
Splicing region	17	0.15	27	0.13
Missense	50	0.43	66	0.32
Total	11,696	100	20,798	100

Within a given breed, QTL for multiple production traits or udder traits were sometimes located in the same genomic region. When we grouped QTL based on their location on the genome, the 84 QTL corresponded to 61 distinct regions, referred to as QTL ID in the first column of Tables 5 and 6. Of these, 36 regions had effects on production traits and 25 had effects on udder morphology and/or health traits. With respect to production traits, the 36 distinct genomic regions corresponded to 53 QTL, which were located on *Bos taurus* (BTA) autosomes 3, 4, 5, 6, 11, 14, 15, 16, 19, 20, 22, 27, and 29 (Table 5). The 25 regions (31 QTL) with effects on udder morphology and health were found on BTA1, 2, 4, 5, 6, 8, 9, 14, 17, 19, 20, 24, 26, 28, and 29 (Table 6). The largest numbers of QTL were found on BTA5, 6, 14, 20, and 19. In addition, 15 of the 61 genomic regions had effects on more than one trait (corresponding to 38 of the 84 QTL); however, no genomic region had pleiotropic effects on both production and udder morphology or health traits (i.e. there was no overlap between the CI of QTL for production traits and any of the other traits). Instead, there were 10 regions that each affected from two to five different production traits and five regions that affected two to three different udder morphology or health traits. Within a breed, even if more than one trait could be linked to a single region, the specific variants with the most significant effects on each trait were largely different. However, there were some cases in which a single variant, always located within a gene, had significant effects on different traits, i.e. the variant with the most significant effects was the same for multiple traits.

**Table 5:** QTL detected for milk production traits in Montbéliarde (MON), Normande (NOR), and Holstein (HOL) bulls

QTL ID^a^	BTA	Breed	Traits (# QTL^b^)	CI of the TOP-CI^c^		Variant with the most significant effect							CI of the EXT-CI^f^	
				Bounds (Mbp)	# Genes	Position (bp)	Variant ID	Functional annotation	MAF	–log_10_(P)	b^e^	sd	Bounds (Mbp)	# Genes
1	3	MON	PC(1)	13.8–15.0	10	14,278,689	rs443751026	*GPATCH4* upstream	0.02	9.2	− 0.667	0.108	13.8–15.0	10
2	3	HOL	PC(2)	15.3–15.9	16	15,540,709	rs207616487	Intergenic	0.03	23.3	− 0.461	0.046	13.6–15.9	19
3	3	NOR	PC(1)	14.7–15.6	6	15,558,071	rs135107424	Intergenic	0.31	12.7	− 0.278	0.038	14.7–15.6	6
4	4	NOR	PC(1)	77.8–79.2	8	78,878,576	rs209194403	Intergenic	0.12	8.6	0.307	0.052	77.8–79.2	8
5	5	MON	FC(1)	85.0–86.8	1	85,867,738	rs378424416	48719* intron	0.03	9.2	− 0.53	0.086	85.0–86.8	1
6	5	HOL	PY(1)	88.6–89.2	5	89,037,941	rs441042365	*GYS2* intron	0.05	8.3	− 0.273	0.047	88.6-89.2	5
7	5	MON	FC(3)	93.7–94.0	2	93,912,041	rs208512412	Intergenic	0.13	10.7	− 0.318	0.048	89.7–94.0	5
8	5	HOL	FC(6)	93.8–94.0	2	93,944,234	rs210886822	*MGST1* intron	0.44	26.4	0.242	0.022	90.8–98.2	13
8	5	HOL	FY(1)	93.9–94.1	2	93,954,751	rs209288972	*MGST1* upstream	0.214	12.3	0.1883	0.026	93.9–94.1	2
9	5	NOR	FC(4)	93.9–94.2	1	94,159,666	rs384934968	Intergenic	0.16	13.6	− 0.335	0.044	89.1–94.2	7
10	5	NOR	MY(1)	112.2–112.5	3	112,281,738	rs110231840	*MRTFA* intron	0.07	8.3	− 0.389	0.066	112.2–112.5	3
11	5	MON	PC(3)	117.1–118.6	8	118,007,875	rs474313622	*GRAMD4* intron	0.04	18.9	− 0.683	0.075	117.1–121.1	15
12	5	HOL	PC(1)	117.8–119.1	3	118,283,121	rs483019568	*TBC1D22A* intron	0.2	11.3	− 0.167	0.024	117.8–119.1	3
13	6	HOL	FC(1)	37.5–38.5	6	37,742,024	rs458142329	*HERC6* intron	0.01	9.7	− 0.442	0.069	37.5–38.5	6
13	6	HOL	PC(1)	37.5–38.1	5	37,742,024	rs458142329	*HERC6* intron	0.012	24.3	− 0.701	0.068	37.5–38.1	5
14	6	HOL	PC(7)	86.0–87.6	4	87,001,138	rs109613975	*38214*^*d*^ intron	0.11	24.1	0.448	0.043	81.7–90.3	18
14	6	HOL	PY(1)	86.9–88	7	87,174,212	rs110217592	Intergenic	0.313	9.6	− 0.156	0.025	86.9–88	7
15	6	NOR	PC(8)	86.5–87.6	4	87,172,459	rs109761275	Intergenic	0.5	22.1	− 0.399	0.041	78.9–91.0	15
16	6	MON	PC(10)	86.6–88.4	8	87,592,824	rs386045066	Intergenic	0.29	23.1	0.411	0.041	78.3–91.2	17
17	6	MON	PC(1)	92.4–94.0	4	93,148,097	rs132964769	SHROOM3 intron	0.43	8.9	0.224	0.037	92.4–94	4
18	6	NOR	PC(1)	92.4–93.5	4	93,231,665	rs43475602	SHROOM3 intron	0.25	9.5	0.32	0.051	92.4–93.5	4
19	11	HOL	FC(1)	103.2–103.4	3	103,294,772	rs207607053	Intergenic	0.48	10.2	0.13	0.02	103.2–103.4	3
20	14	NOR	FC(5)	1.5–2.0	19	1,800,399	rs208317364	*DGAT1* intron/HSF1 downstream	0.09	89.0	1.04	0.052	1.5–9.3	33
20	14	NOR	FY(1)	1.7–2	19	1,810,124	rs133931291	*HSF1* intron	0.09	14.3	0.4301	0.055	1.7–2	19
20	14	NOR	PC(1)	1.5–2	19	1,800,399	rs208317364	*DGAT1* intron/HSF1 downstream	0.094	14.5	0.417	0.053	1.5–2	19
21	14	MON	FC(7)	1.4–2.0	23	1,881,116	rs132649038	*MROH1* intron/bta-mir-1839 downstream	0.02	62.4	1.935	0.115	1.4–10.5	45
21	14	MON	MY(1)	1.4–2.3	24	2,261,103	rs517360248	*ZNF623* downstream	0.169	9.9	− 0.239	0.037	1.4–2.3	24
21	14	MON	FY(1)	1.4–2.3	24	2,261,103	rs517360248	*ZNF623* downstream	0.169	9.9	− 0.239	0.037	1.4–2.3	24
22	14	HOL	FC(5)	1.8–3.0	19	1,881,400	rs210517654	*MROH1* intron/bta-mir-1839 downstream	0.2	295.1	0.916	0.022	1.5–9.7	55
22	14	HOL	MY(5)	1.5–2.1	29	1,801,116	rs109421300	*DGAT1* intron/HSF1 downstream	0.203	100.8	− 0.49	0.023	1.5–7.1	29
22	14	HOL	FY(6)	1.5–2.2	29	1,801,116	rs109421300	*DGAT1* intron/HSF1 downstream	0.203	128.2	0.5803	0.024	1.5–9.7	29
22	14	HOL	PY(2)	1.5–2.1	31	1,801,116	rs109421300	*DGAT1* intron/HSF1 downstream	0.203	22.3	− 0.231	0.023	1.5–3.0	31
22	14	HOL	PC(3)	1.5–2.2	25	1,801,116	rs109421300	*DGAT1* intron/HSF1 downstream	0.203	121.1	0.5048	0.022	1.5–4.5	25
23	14	MON	FC(1)	12.4–12.8	0	12,601,610	rs207790129	Intergenic	0.04	11.5	0.583	0.084	12.4–12.8	0
24	14	HOL	FC(3)	65.4–67.4	6	66,419,482	rs211058631	*FBXO43* upstream/POLR2K upstream	0.11	13.3	− 0.257	0.034	65.3–67.5	6
24	14	HOL	MY(3)	65.3–67.4	6	66,326,942	rs110125070	*SPAG1* intron	0.147	13.4	0.2125	0.028	64.5–68.1	6
24	14	HOL	PC(5)	65.3–67.4	6	66,326,942	rs110125070	*SPAG1* intron	0.147	35.7	− 0.334	0.027	63.6–69.7	6
25	15	HOL	PC(1)	27.9–29.2	10	28,802,897	rs207905326	Intergenic	0.21	8.4	0.153	0.026	27.9–29.2	10
26	16	MON	PC(1)	60.3–60.9	1	60,618,083	rs43317278	Intergenic	0.34	8.7	0.194	0.032	60.3–60.9	1
27	19	HOL	FC(1)	51.3–51.4	2	51,321,632	rs109042366	*CCDC57* intron	0.32	14.0	0.174	0.022	51.3–51.4	2
27	19	HOL	FY(1)	51.3–51.4	2	51,323,848	rs41921170	*CCDC57* intron	0.318	10.9	0.1618	0.024	51.3–51.4	2
28	20	HOL	FY(1)	7.5–7.8	1	7661,288	rs467849681	*ARHGEF28* intron	0.02	8.6	0.341	0.057	7.5–7.8	1
28	20	HOL	PY(1)	7.5–7.7	1	7339,763	rs455099807	Intergenic	0.001	8.3	1.0799	0.185	7.5–7.7	1
29	20	HOL	PC(1)	29.3–30.1	1	30,031,902	rs211443146	Intergenic	0.22	11.7	− 0.199	0.028	29.3–30.1	1
30	20	HOL	PC(3)	31.2–33.2	6	32,254,539	rs209333496	Intergenic	0.2	29.6	− 0.357	0.031	31.1–34.3	16
30	20	HOL	MY(1)	31.2–33.2	5	32,265,342	rs41943564	Intergenic	0.197	8.2	0.1864	0.032	31.2–33.2	5
30	20	HOL	FC(3)	31.3–32.6	2	32,296,239	rs210730645	Intergenic	0.201	17.4	− 0.274	0.032	31.1–34.3	2
31	22	MON	PC(1)	32.7–33.0	1	32,779,678	rs109419324	*TAFA4* intron	0.01	8.5	− 0.701	0.118	32.7–33.0	1
32	27	MON	FC(1)	36.1–36.4	4	36,206,783	rs210205723	Intergenic	0.45	8.4	− 0.201	0.034	36.1–36.4	4
33	27	HOL	FC(1)	36.1–36.3	3	36,221,754	rs208624037	*GPAT4* intron	0.38	11.1	− 0.149	0.022	36.1–36.3	3
34	27	NOR	FC(1)	36.1–36.4	3	36,301,028	rs383292923	*ANK1* intron	0.34	9.1	− 0.197	0.032	36.1–36.4	3
35	29	HOL	PC(1)	9.0–9.7	2	9563,396	rs378183369	Intergenic	0.31	12.6	0.158	0.022	9.0–9.7	2
36.	29	HOL	PC(2)	41.4–43.4	12	42,393,898	rs211552605	*LGALS12* upstream	0.15	11.6	− 0.226	0.032	41.4–44.2	30

#: number of

^a^ID number associated with a group of QTL linked with milk production traits that had overlapping confidence intervals or less than 1 Mbp distance between the bounds of the confidence intervals

^b^Individual QTL; milk yield (MY), fat content (FC), protein content (PC), fat yield (FY), protein yield (PY)

^c^Confidence interval (CI) of the most significant individual QTL

^d^ENSBTAG000000

^e^Effect of the variant, expressed in genetic standard deviation units

^f^Extended confidence interval (CI) encompassing all the individual QTL detected in the same genomic region

**Table 6:** QTL detected for udder traits in Montbéliarde (MON), Normande (NOR), and Holstein (HOL) bulls

QTL ID^a^	BTA	Breed	Traits (# QTL^b^)	CI of the TOP-CI^c^		Variant with the most significant effect							CI of the EXT-CI^f^	
				Bounds (Mbp)	# Genes	Position (bp)	Variant ID	Functional annotation	MAF	− log_10_(P)	b^e^	sd	Bounds (Mbp)	# genes
37	1	HOL	US(1)	110.3–111.0	1	110,870,166	rs42324415	Intergenic	0.32	9.3	0.19	0.031	110.3–111.0	1
38	2	NOR	RUH(4)	6.0–6.7	2	6,237,254	rs385990556	Intergenic	0.02	16.5	− 1.185	0.14	4.5–9.2	11
39	4	HOL	UD(1)	10.0–10.2	0	10,126,995	rs42524022	Intergenic	0.36	8.5	0.131	0.022	10.0–10.2	0
40	4	HOL	FTD(1)	70.6–70.7	0	70,620,943	rs210515134	Intergenic	0.47	8.9	− 0.182	0.03	70.6–70.7	0
41	5	HOL	TL(1)	12.4–12.5	1	12,441,072	rs109808447	TMTC2 Intron	0.14	25	0.379	0.036	12.4–12.5	1
42	5	HOL	UD(1)	88.7–88.9	2	88,800,994	rs110461240	*ABCC9* intron	0.45	12.4	0.186	0.026	88.7–88.9	2
42	5	HOL	FUA(1)	88.7–88.9	2	88,800,994	rs110461240	*ABCC9* intron	0.457	11.3	0.1787	0.026	88.7–88.9	2
43	6	HOL	RUH(1)	71.9–72.2	0	72,022,013	rs136970036	Intergenic	0.28	14.9	0.272	0.034	71.9–72.2	0
44	6	MON	UD(1)	88.6–89.6	1	88,744,985	rs110181141	Intergenic	0.37	9.2	− 0.256	0.042	88.6–89.6	5
45	6	HOL	SCS(1)	88.4–89.2	3	88,881,928	rs474137839	Intergenic	0.31	9.2	0.206	0.033	88.4–89.2	3
45	6	HOL	UD(1)	88.4–89.2	4	88,697,293	rs110041776	*GC* intron	0.407	8.6	0.1789	0.03	88.4–89.2	4
46	8	HOL	FTD(2)	81.9–83.1	3	82,059,790	rs134625094	Intergenic	0.11	10.8	− 0.316	0.047	81.9–85.4	8
47	9	MON	SCS(1)	23.4–23.7	1	23,620,280	rs437752750	ME1 Intron	0.02	9.5	− 0.718	0.114	23.4–23.7	1
48	9	MON	SCS(1)	25.7–26.3	1	25,888,225	rs797068196	*HEY2* upstream	0.02	9.1	− 0.78	0.127	25.7–26.3	1
49	9	MON	SCS(1)	28.1–29.2	1	28,197,098	rs455285205	*38849*^d^ intron	0.01	8.2	− 0.859	0.147	28.1–29.2	1
50	14	MON	RUH(1)	24.4–25.1	3	25,052,440	rs210030313	*PLAG1* 5′UTR*/CHCHD7* upstream	0.07	12.5	− 0.506	0.07	24.4–25.1	3
51	17	MON	FUA(1)	62.6–62.7	0	62,679,331	rs109168890	Intergenic	0.42	13.1	− 0.283	0.038	62.6–62.7	0
51	17	MON	US(1)	62.6–62.9	0	62,714,882	rs109332098	Intergenic	0.215	12.3	− 0.358	0.049	62.6–62.9	0
52	17	NOR	US(1)	62.5–62.9	2	62,694,032	rs109134926	Intergenic	0.21	10.0	− 0.272	0.042	62.5–62.9	2
53	19	HOL	MSS(1)	7.5–7.7	1	7,565,865	rs445450010	Intergenic	0.47	10.5	− 0.153	0.023	7.5–7.7	1
54	19	HOL	MSS(1)	59.3–60.4	0	60,151,690	rs109603247	Intergenic	0.18	8.9	0.186	0.031	59.3–60.4	0
55	19	MON	UD(1)	60.5–61.5	0	60,523,834	rs134785404	Intergenic	0.37	9.0	− 0.199	0.032	60.5–61.5	0
55	19	MON	MSS(1)	59.3–60.4	0	60,151,690	rs109603247	Intergenic	0.183	8.9	0.1856	0.031	59.3–60.4	0
56	20	HOL	FUA(3)	26.1-28.0	2	27,082,437	rs209792391	Intergenic	0.29	10.9	− 0.192	0.028	25.6––28	3
57	24	MON	UB(1)	33.2–35.3	11	34,288,737	rs382921722	Intergenic	0.4	9.1	0.326	0.053	33.2–35.3	11
58	24	HOL	RUH(1)	33.3–34.6	3	34,310,163	rs482718265	Intergenic	0.04	10.5	− 0.377	0.057	33.3–34.6	3
59	26	HOL	TO(1)	38.5–38.9	2	38,725,076	rs133440951	Intergenic	0.05	8.6	− 0.302	0.051	38.5–38.9	2
60	28	MON	MSS(1)	19.3–21.1	4	20,158,547	rs43101108	Intergenic	0.09	8.7	− 0.342	0.057	19.3–21.1	4
61	29	HOL	UD(3)	49.1–50.3	4	49,421,004	rs208859984	KCNQ1 Intron	0.31	12.7	− 0.167	0.023	49.1–51.5	24
61	29	HOL	SCS(1)	49.3–50.3	4	49,782,986	rs437410319	KCNQ1 Intron	0.066	8.4	0.2493	0.042	49.3–50.3	4
61.	29	HOL	UB(2)	48.3–50.3	8	49,299,622	rs210749543	NAP1L4 Intron	0.052	10.8	− 0.347	0.051	48.3–51.2	8

#: number of

^a^ID number associated with a group of QTL linked with udder traits that had overlapping confidence intervals or less than 1 Mbp distance between the bounds of the confidence intervals

^b^Individual QTL; somatic cell score (SCS), clinical mastitis score (CM), udder support (US), udder depth (UD), fore udder attachment (FUA), rear udder height (RUH), fore teat distance (FTD), udder balance (UB), teat orientation (TO), teat length (TL), and milking speed score (MSS)

^c^confidence interval (CI) of the most significant individual QTL

^d^ENSBTAG000000

^e^Effect of the variant, expressed in genetic standard deviation units

^f^Extended confidence interval (CI) encompassing all the individual QTL detected in the same genomic region

We identified 15 QTL ID, all linked with production traits, that were shared among the three breeds; they were located in five genomic regions and affected PC on BTA3 (at ~ 15 Mbp) and BTA6 (at ~ 87 Mbp, Fig. 2) and FC on BTA5 (at ~ 94 Mbp), BTA14 (at ~ 1.8 Mbp), and BTA27 (at ~ 36.2 Mbp). Six other regions (two for production traits and four for udder traits) had effects in two different breeds: in HOL and NOR on BTA5 (at ~ 118 Mbp for PC); in HOL and MON on BTA6 (at ~ 88.8 Mbp for UD, Fig. 4; at ~ 93 Mbp for PC), BTA19 (at ~ 60 Mbp for MSS and UD), and BTA24 (at ~ 34 Mbp for UB and RUH) and in MON and NOR on BTA17 (at ~ 62.7 Mbp for FUA and US). Although regions were shared among breeds, the variants with the most significant effect were different in each breed, with one exception: in one region located on BTA19, the intergenic variant rs109603247 had the most significant effect on MSS in both HOL and MON.

In all the QTL detected, the size of the TOP-CI ranged from 36.7 kb (BTA4 in HOL for FTD) to 1.9 Mb (BTA24 in MON for UB), with mean and median values being equal to 931 and 700 kb, respectively. TOP-CI contained from 0 to 31 genes (mean = 6.1; median = 3). As expected, EXT-CI were often broader, up to 12.8 Mb (mean = 2.1 Mb; median = 1.0 Mb) with a larger number of genes, up to 55 (mean = 8.5; median = 4). When we analyzed the EXT-CI of QTL, we observed that the majority contained at least a gene; only nine QTL (1 for production and 8 for udder morphology and/or health traits) were located entirely within intergenic regions. The variant with the most significant effect was located in an intergenic region for 19 out of 53 QTL identified for production traits and for 20 out of 31 QTL found for udder traits. All other variants presenting the most significant effects were located in intronic (28 for production traits and 9 for udder traits), upstream (4 for production traits and 1 for udder traits), downstream (2 for production traits and 1 for udder traits) or 5′UTR (1 for production trait) regions of genes. The genes in which these variants were located are indicated in Tables 5 and 6.

### Part II: Confirmation results on cows’ performances

Within each of the 84 QTL detected in Part I, we selected the variants that best explained the observed results, hereafter named candidate variants, from sequence-based GWAS results from bulls. For technical reasons, a few of these candidate variants could not be included on the customized EuroG10K chip. In the end, one to 192 candidate variants from each of 80 of the 84 QTL (855 different variants in total) were added to the chip and tested for validation together with the standard 50K SNPs. As a consequence, even for the four QTL for which no candidate variant was added (two for production and two for udder traits), there were SNPs from the standard 50K chip that were located in the EXT-CI and were thus included in this confirmation study. We confirmed—i.e. found significant effects in the corresponding breed x trait analysis—the effects in cows of 54 out of the 84 QTL described in Tables 5 and 6 (40 of 53 QTL for production traits, Table 7; 14 out of 31 QTL for udder traits, Table 8). In each of the validated QTL regions, we found significant effects (− log_10_(P) ≥ 6) for up to 99 candidate variants and up to 33 50K SNPs. Of the 80 QTL for which we tested candidate variants, the mean rank of the best candidate variant was 1.8 for all the QTL, for both production and udder traits, and 1.5 for the validated QTL (1.6 for production traits and 1.1 for udder traits). Thus, for the majority of the validated QTL, the variant with the most significant effect was one of the candidate variants selected in Part I for its level of significance and/or annotation; the exceptions were seven QTL that corresponded to four different genomic regions. Of these four regions, we found one in which only one candidate variant was present (at ~ 78 Mb on BTA4 for PC in NOR); another one in which the best candidate variant was ranked 2nd (at ~ 12 Mb on BTA5 for TL in HOL); and two regions linked with production traits in HOL, both located on BTA14 (~ 1.8 Mb and ~ 67.4 Mb). The first region on BTA14 (~ 1.8 Mb) had very significant effects on all five production traits, and for three of them (FC, FY, and PC), one of the candidate variants was ranked first in the peak. In contrast, for the second region (~ 68 Mb), the candidate variant with the most significant effects on FC, MY, and PC was ranked 3rd, 4th and 4th, respectively, in the peak, meaning that the top two or three variants were from the set of 50K SNPs. Therefore, for almost all the QTL for which the effects were validated in Part II of this study, candidate variants from Part I had more significant effects than the SNPs from the 50K chip.

**Table 7:** GWAS validation results for production traits in Montbéliarde (MON), Normande (NOR), and Holstein (HOL) cows

QTL ID^a^	BTA	Breed	Traits (# QTL^b^)	Candidate variants^c^				50K SNPs				Variant with the most significant effect			
				Total#	# with -log_10_(P) > 6	# in TOP10	Best rank	Total#	# with -log_10_(P) > 6	# in TOP10	Best rank	Position (pb)	ID	-log_10_(P)	Functional annotation
1	3	MON	PC(1)	8	0	4	1	18	0	6	2	14,889,296	rs110992770	2.7	KHCD4 upstream
2	3	HOL	PC(2)	16	3	4	1	31	3	6	3	15,592,645	rs134511693	66.6	EFNA4 upstream/ADAM15 downstream
3	3	NOR	PC(1)	9	4	6	1	15	2	4	3	15,558,071	rs135107424	16.0	Intergenic
4	4	NOR	PC(1)	1	0	0	15	23	3	10	1	78,114,069	rs42683912	10.6	COA1 intron
5	5	MON	FC(1)	2	0	2	5	22	0	8	1	86,753,605	rs109647158	1.7	SOX5 intron
6	5	HOL	PY(1)	18	0	8	1	9	0	2	4	88,830,128	rs136903701	5.5	ABCC9 intron/downstream
7	5	MON	FC(3)	37	17	10	1	51	3	0	16	93,945,738	rs211210569	40.8	MGST1 intron
8	5	HOL	FC(6)	51	33	10	1	95	16	0	30	93,945,991	rs208248675	120.3	MGST1 intron
8	5	HOL	FY(1)	31	26	10	1	3	0	0	29	93,945,738	rs211210569	25.2	MGST1 intron
9	5	NOR	FC(4)	40	25	10	1	57	4	0	24	93,945,738	rs211210569	26.8	MGST1 intron
10	5	NOR	MY(1)	16	0	8	1	4	0	2	2	112,398,982	rs211569025	2.1	Intergenic
11	5	MON	PC(3)	22	9	7	1	75	10	3	2	117,972,265	rs525880746	68.6	GRAMD4 upstream
12	5	HOL	PC(1)	5	1	1	1	32	1	9	2	118,244,695	rs456403270	23.5	TBC1D22A missense
13	6	HOL	FC(1)	8	0	3	1	21	0	7	3	37,723,413	rs136548039	2.9	HERC5 intron
13	6	HOL	PC(1)	6	0	3	3	13	0	7	1	38,063,313	rs41622323	4.4	PKD2 intron
14	6	HOL	PC(7)	174	89	10	1	104	20	0	27	87,199,843	rs383909572	56.0	HSTN splice acceptor
14	6	HOL	PY(1)	87	35	8	1	18	2	2	5	87,181,619	rs43703011	14.6	CSN2 missense
15	6	NOR	PC(8)	192	99	10	1	158	23	0	33	87,296,809	rs134776019	25.3	Intergenic
16	6	MON	PC(10)	190	92	9	1	164	29	1	2	87,296,809	rs134776019	54.4	Intergenic
17	6	MON	PC(1)	10	0	2	4	30	0	8	1	92,623,916	rs41256838	2.1	CXCL10 5′UTR/ART3 intron
18	6	NOR	PC(1)	8	3	6	1	23	3	4	2	92,561,862	rs133076983	6.8	SDAD1 missense
19	11	HOL	FC(1)	31	30	10	1	4	1	0	28	103,300,548	rs109982707	26.3	PAEP upstream
20	14	NOR	FC(5)	73	44	13	1	188	15	0	14	1,891,657	rs109136389	137.6	MROH1 intron
20	14	NOR	FY(1)	39	24	20	1	3	3	1	2	1,808,145	rs135258919	10.2	HSF1 missense/DGAT1 downstream
20	14	NOR	PC(1)	43	41	14	1	7	4	1	2	1,881,116	rs132649038	26.4	MROH1 intron/bta-mir-1839 downstream
21	14	MON	FC(7)	61	22	9	1	224	27	1	10	1,795,176	rs379230475	129.4	DGAT1 5′UTR/SCRT1 downstream
21	14	MON	MY(1)	17	15	10	1	13	5	0	12	1,795,176	rs379230475	18.0	DGAT1 5′UTR/SCRT1 downstream
21	14	MON	FY(1)	17	10	10	1	13	0	0	11	1,639,005	rs384162250	8.8	Intergenic
22	14	HOL	FC(5)	90	52	37	1	206	33	2	1	1,802,265	rs109234250	>300	DGAT1 missense
22	14	HOL	MY(5)	72	48	11	2	139	24	1	1	1,801,116	rs109421300	108.1	DGAT1 intron/HSF1 downstream
22	14	HOL	FY(6)	90	50	10	1	206	26	0	19	1,739,885	rs110825388	93.7	CPSF1 intron/ADCK5 downstream
22	14	HOL	PY(2)	53	45	11	2	30	8	1	1	1,801,116	rs109421300	30.3	DGAT1 intron
22	14	HOL	PC(3)	62	48	13	1	64	18	0	20	1,724,688	rs135458711	141.9	SLC39A4 downstream/CPSF1 upstream
23	14	MON	FC(1)	0	0	0	0	10	0	10	1	12,672,880	rs42381926	1.6	Intergenic
24	14	HOL	FC(3)	1	1	1	3	27	10	9	1	67,443,766	rs109007040	11.9	VPS13B intron
24	14	HOL	MY(3)	4	2	2	4	51	4	8	1	67,443,766	rs109007040	6.2	VPS13B intron
24	14	HOL	PC(5)	5	3	2	4	90	23	8	1	67,443,766	rs109007040	31.0	VPS13B intron
25	15	HOL	PC(1)	0	0	0	0	27	0	10	1	27,905,645	rs110144962	2.5	APOA4 downstream
26	16	MON	PC(1)	1	1	1	1	13	0	9	2	60,692,234	rs135698521	7.9	Intergenic
27	19	HOL	FC(1)	11	9	9	1	3	1	1	4	51,319,797	rs41921161	16.6	CCDC57 missense
27	19	HOL	FY(1)	11	0	9	1	3	0	1	4	51,319,797	rs41921161	5.9	CCDC57 missense
28	20	HOL	FY(1)	5	0	3	4	7	0	7	1	7,661,649	rs110231369	0.5	ARHGEF28 intron
28	20	HOL	PY(1)	4	0	4	3	5	0	5	1	7,661,649	rs110231369	0.8	ARHGEF28 intron
29	20	HOL	PC(1)	1	1	1	1	11	2	9	2	30,005,528	rs43140727	11.3	Intergenic
30	20	HOL	PC(3)	103	92	10	1	48	12	0	50	31,909,478	rs385640152	102.4	GHR missense
30	20	HOL	MY(1)	80	37	14	1	23	0	0	41	31,303,953	rs474736745	9.9	PAIP1 intron
30	20	HOL	FC(3)	103	82	10	1	48	6	0	46	31,909,478	rs385640152	54.5	GHR missense
31	22	MON	PC(1)	3	0	3	3	4	1	4	1	32,827,786	rs41642478	1.0	TAFA4 intron
32	27	MON	FC(1)	8	4	6	1	7	2	4	5	36,209,319	rs211250281	20.4	GPAT4 upstream
33	27	HOL	FC(1)	5	4	5	1	6	4	5	5	36,212,352	rs208675276	33.3	GPAT4 5′ UTR
34	27	NOR	FC(1)	8	4	7	1	8	2	4	5	36,211,258	rs209479876	14.7	GPAT4 upstream
35	29	HOL	PC(1)	4	2	3	1	15	1	7	2	9,608,833	rs378017490	10.0	PICALM upstream
36.	29	HOL	PC(2)	50	5	5	1	56	4	5	4	41,843,734	rs208817293	15.9	WDR74 intron/U2 downstream

#: number of

^a^ID number associated with a group of QTL linked with milk production traits that had overlapping confidence intervals or less than 1 Mbp distance between the bounds of the confidence intervals

^b^Individual QTL; milk yield (MY), fat content (FC), protein content (PC), fat yield (FY), protein yield (PY)

^c^Candidate variants selected from sequence-based GWAS results

**Table 8:** GWAS validation results for udder traits in Montbéliarde (MON), Normande (NOR), and Holstein (HOL) cows

QTL ID^a^	BTA	Breed	Traits (# QTL^b^)	Candidate variants^c^				50K SNPs				Variant with the most significant effect			
				# Total	# with -log_10_(P) > 6	# in TOP10	Best rank	Total#	# with -log_10_(P) > 6	# in TOP10	Best rank	Position (pb)	ID	− log_10_(P)	Functional annotation
37	1	HOL	US(1)	4	2	4	1	11	0	6	3	110,870,166	rs42324415	6.8	Intergenic
38	2	NOR	RUH(4)	14	0	2	1	80	0	8	2	6,325,829	rs385535384	3.0	Intergenic
39	4	HOL	UD(1)	0	0	0	0	3	0	3	1	10,078,207	rs42524753	1.7	Intergenic
40	4	HOL	FTD(1)	5	0	5	3	2	0	2	1	70,680,040	rs41652994	1.0	Intergenic
41	5	HOL	TL(1)	5	4	5	2	3	1	3	1	12,443,146	rs41604034	10.7	TMTC2 intron
42	5	HOL	UD(1)	17	8	9	1	3	1	1	4	88,812,245	rs209585944	16.9	ABCC9 intron
42	5	HOL	FUA(1)	17	8	9	1	3	1	1	3	88,812,245	rs209585944	17.6	ABCC9 intron
43	6	HOL	RUH(1)	0	0	0	0	18	0	7	1	72,028,756	rs110103615	3.8	Intergenic
44	6	MON	UD(1)	20	17	8	1	7	0	2	7	88,723,742	rs436532576	19.1	GC intron
45	6	HOL	SCS(1)	37	6	10	1	14	3	0	18	88,723,742	rs436532576	6.3	GC intron
45	6	HOL	UD(1)	37	22	10	1	19	0	0	18	88,723,742	rs436532576	19.4	GC intron
46	8	HOL	FTD(2)	4	0	3	3	19	0	7	1	83,693,221	rs41570498	3.5	Intergenic
47	9	MON	SCS(1)	2	0	2	2	12	0	5	1	23,517,815	rs41611219	2.7	ME1 intron
48	9	MON	SCS(1)	1	0	1	7	12	5	9	1	25,793,691	rs109661188	0.3	NCOA7 intron
49	9	MON	SCS(1)	1	0	1	1	13	0	9	2	29,130,097	.	0.8	HSF2 missense
50	14	MON	RUH(1)	24	12	10	1	17	0	0	12	25,015,640	rs109815800	29.5	PLAG1 intron
51	17	MON	FUA(1)	3	0	3	1	60	0	2	4	62,695,902	rs109014048	5.9	Intergenic
51	17	MON	US(1)	9	8	9	1	6	0	1	10	62,809,661	rs110701343	6.9	Intergenic
52	17	NOR	US(1)	10	5	8	1	5	0	2	6	62,693,355	rs109184112	14.7	Intergenic
53	19	HOL	MSS(1)	12	12	9	1	11	0	1	9	7,543,195	rs109941836	8.5	Intergenic
54	19	HOL	MSS(1)	11	0	2	3	18	0	8	1	60,009,650	rs41653204	3.3	Intergenic
55	19	MON	UD(1)	4	0	4	3	6	1	6	1	61,478,388	rs109108437	5.4	Intergenic
55	19	MON	MSS(1)	10	0	7	3	2	0	3	1	59,984,238	rs110395313	3.1	Intergenic
56	20	HOL	FUA(3)	13	4	4	1	5	0	6	2	27,756,459	rs382512825	10.1	ISL1 upstream
57	24	MON	UB(1)	13	8	8	1	8	2	2	9	34,317,850	rs210449055	13.7	Intergenic
58	24	HOL	RUH(1)	17	0	9	1	5	1	1	5	34,317,850	rs210449055	2.7	Intergenic
59	26	HOL	TO(1)	8	8	8	1	26	0	2	9	38,617,279	rs109011767	10.7	RAB11FIP2 3′ UTR
60	28	MON	MSS(1)	2	0	1	3	15	1	9	1	20,014,813	rs42137452	2.2	Intergenic
61	29	HOL	UD(3)	20	0	2	3	27	0	8	1	49,412,703	rs42194458	1.9	KCNQ1 intron
61	29	HOL	SCS(1)	11	0	4	1	25	5	6	2	50,066,017	rs133306466	2.2	IGF2 downstream
61	29	HOL	UB(2)	23	0	6	2	43	0	4	1	50,296,573	rs42196507	2.8	SYT8 upstream

#: number of

^a^ID number associated with a group of QTL linked with udder traits that had overlapping confidence intervals or less than 1 Mbp distance between the bounds of the confidence intervals

^b^Individual QTL; somatic cell score (SCS), clinical mastitis score (CM), udder support (US), udder depth (UD), fore udder attachment (FUA), rear udder height (RUH), fore teat distance (FTD), udder balance (UB), teat orientation (TO), teat length (TL), and milking speed score (MSS); ^c^ candidate variants selected from sequence-based GWAS results

**Fig. 5:**
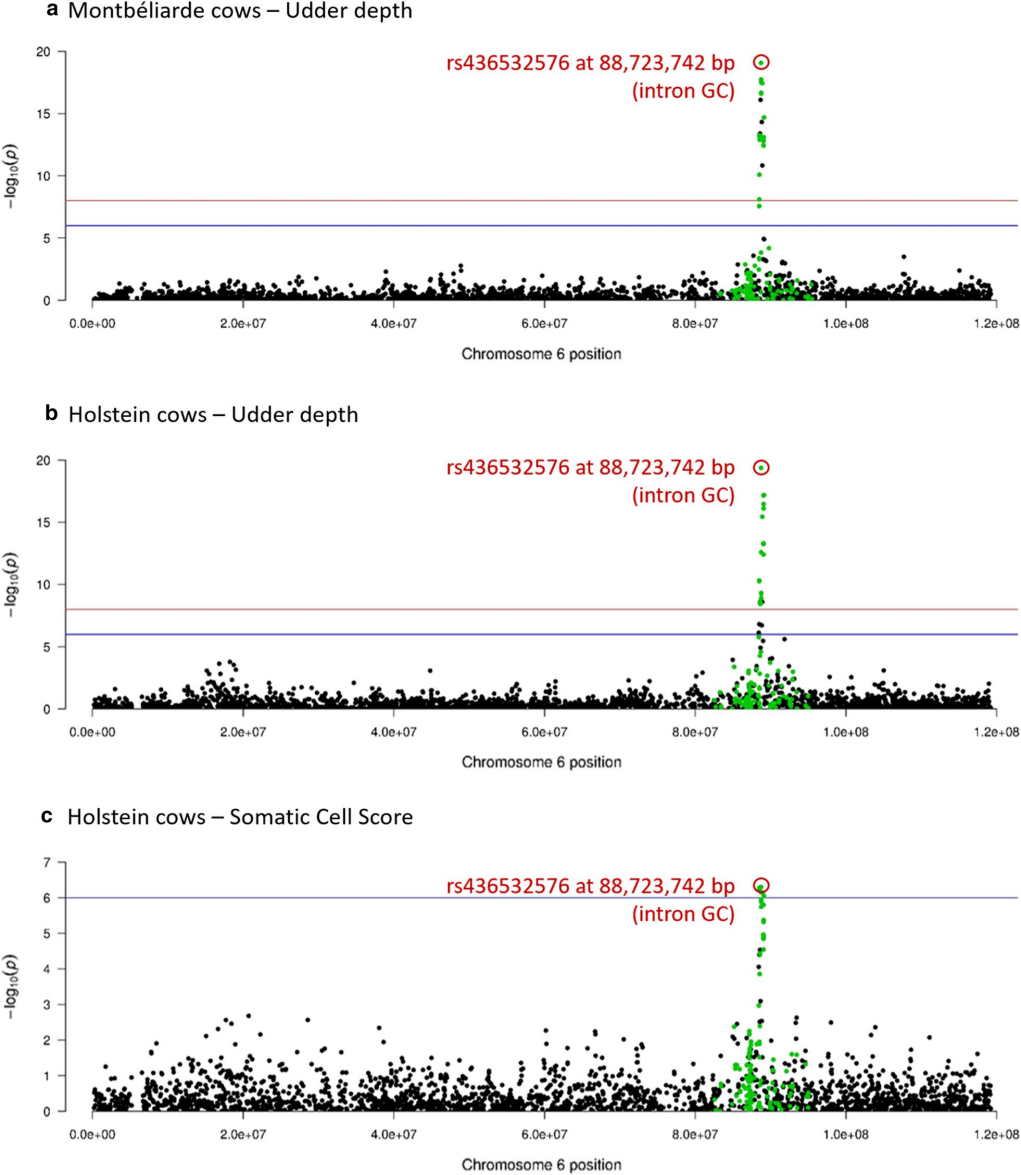
Validation GWAS: –log_10_(P) plotted against the position on *Bos taurus* chromosome 6 of variants linked with udder depth in **a** Montbéliarde and **b** Holstein cows, and **c** with somatic cell score (SCS) in Holstein cows; in black, 50K SNPs; in green, candidate variants; red circle indicates the variant with the most significant effect

In 47 of the validated QTL, a candidate variant from Part I presented the most significant effects; these corresponded to 39 unique variants. Six of these had the most significant effects on two or three different traits and/or different breeds. In particular, we identified three candidate variants having the most significant effects in different breeds: an intronic variant in the *MGST1* gene (rs211210569) for FC in all three breeds; an intergenic variant on BTA6 (rs134776019) for PC in MON and NOR; and an intronic variant in the *GC* gene (rs436532576) for SCS or UD in HOL and MON (Fig. 5). Two additional variants presented the most significant effects on different traits within a single breed: the rs379230475 variant, located in the 5′UTR region of *DGAT1* (BTA14), was the top variant for FC and MY in MON and the missense rs385640152 variant in *GHR* (BTA 20) was the top variant for PC and FC in HOL. In most of the genomic regions for which effects were observed in different breeds or traits, the variants that had the most significant effects were distinct, and multiple variants were located in the same gene in only a few cases (*MGST1*, *DGAT1*, and *GPAT4*). Of the 39 variants with the most significant effects, 10 were in intergenic regions (5 for production traits and 5 for udder traits) while 29 were located in genes listed in Tables 7 and 8.

## Discussion

The approach used in Part I of this study—GWAS on imputed whole genome sequences in bulls and the selection of candidate variants in QTL regions—led to the identification of 84 QTL for traits related to production (53), udder morphology (26), and udder health (5) in the three main French dairy breeds. In Part II, we investigated these QTL in statistically independent populations of cows, and confirmed the effects of 54 of them (40 of 53, 75%, for production traits and 14 of 31, 45%, for udder traits). In addition, by performing a GWAS with sequence-level resolution on thousands of bulls for which accurate phenotypes were available, we were able to propose 855 candidate causative variants in the QTL regions, of which 452 were validated in large populations of cows (9400 to 51,977, depending on the breed).

However, the number of QTL detected and validated differed between breeds. The sequence-based GWAS identified twice as many QTL in HOL (48) as in MON (24), and four times more than in NOR (12). Likewise, the proportion of validated QTL was also higher in HOL than in MON or NOR (32, 12, and 10 QTL, respectively). Furthermore, regardless of the breed, both the number of QTL and their level of significance varied among traits. In the sequence-based GWAS, the 84 QTL corresponded to 36 different genomic regions linked with production traits (mean − log_10_(P) value of 27.1), 23 regions associated with udder morphology (mean − log_10_(P) value of 11.2), and five regions for udder health traits (mean − log_10_(P) value of 8.9).

### Factors that can affect GWAS results

The differences that we observed in GWAS results may, at least in part, have been due to factors that were unique to the breeds, traits, and populations (bulls and cows) analyzed here.

#### Number of animals with phenotypes and genotypes

HOL, MON, and NOR cows represent 64, 19, and 9% of French dairy herds, respectively. For this reason, the number of animals with phenotypes was much larger in HOL than in MON or NOR (6262 HOL bulls vs 2434 MON and 2175 NOR for the primary detection; 51,977 HOL cows vs. 23,926 MON and 9400 NOR for the validation). This discrepancy clearly affected the power of detection in both sets of analyses: we were able to detect and validate QTL with smaller effects in the HOL population, and consequently, identified more QTL in total in HOL than in the other two breeds.

#### Imputation accuracies

The number of sequenced bulls included in RUN4 of the 1000 Bull Genomes Project, and therefore in the reference population for sequence-level imputation, were 288, 28, and 24 in HOL, MON, and NOR, respectively. Unsurprisingly, the estimated imputation accuracies were then higher in HOL than in MON [19] and NOR. In addition, MON is related to the Simmental breed that is well represented in the 1000 bull genome population, whereas NOR is quite specific and likely benefits less from the sequences of the other breeds. In addition, we estimated that the 28 MON and 24 NOR bulls whose sequences were included in the reference population had cumulative contributions to the French populations of 64 and 59%, respectively. These differences may have also promoted a higher imputation accuracy in MON than in NOR and therefore explain the smaller number of QTL found for the NOR bulls.

Between the bull and cow populations, missing genotypes were imputed based on different reference populations. For imputations of bull genotypes (WGS), we used a multi-breed reference population that consisted of their major ancestor bulls. This reference population was of limited size, especially within breed, which likely affected the accuracy of imputation, especially for breed-specific and/or low-MAF variants. For imputations of cow genotypes (50K SNPs + candidate variants), we used large within-breed reference populations that consisted of all animals genotyped with the EuroG10k chip; thus, imputation accuracy was much higher than that at the sequence level.

#### Heritability and reliability of traits

Differences among traits in the numbers of QTL detected in the sequence-based GWAS could also be explained by differences in DYD reliabilities. DYD is considered as a bull’s own performance for a trait, the heritability of which would be equal to the reliability of the DYD value. The higher the reliability, the smaller the residual variance and the higher the detection power. In addition to the heritability of the trait, the reliability of the DYD also depends on the effective daughter contribution [21], and on average, progeny groups were a little larger in HOL than in MON and NOR. Because udder health traits had lower heritabilities (h^2^ = 0.018 to 0.15), the reliability (REL) of their DYD values was lower (REL = 0.40 to 0.88) than for udder morphology traits (h^2^ = 0.15 to 0.45; REL = 0.74 to 0.95) and production traits (h^2^ = 0.30 to 0.50; REL = 0.89 to 0.95) (Tables 2 and Additional file 1: Table S1). In addition, morphological traits were recorded only once for each daughter, whereas DYD calculations for production and health traits included up to three lactations per cow. Finally, recording of CM started only recently and is not exhaustive [22], meaning that DYD information is available for fewer bulls, with smaller informative progeny groups. All these reasons explain why the power of detection decreased from analyses of milk composition to those of milk yield, udder type, SCS, and finally CM.

In the cow confirmation study, the sample size was larger than in the bull populations, but the reliability of the traits, equal to the heritability (for non-repeated records), was always lower than reliability of the DYD, and for CM, considerably so. Depending on the trait and the population in question, the power of detection in the cow populations was either higher (e.g., for HOL and MON and high or medium heritability traits) or lower (e.g., for CM). The resulting lower power of the validation dataset in some cases could be a possible explanation why certain variant effects were unconfirmed.

For these reasons, we were able to explain a higher percentage of genetic variance for the most heritable traits and to detect small QTL for the phenotypes with the highest accuracy. Regardless of the breed analyzed, our results varied widely among traits. We detected no QTL for CM, the trait with the lowest values of heritability (≤ 0.023) and reliability (≤ 0.43), while for FC and PC, which had the highest heritability (0.50) and reliability (0.92–0.94), we recovered the largest number of QTL (up to 11 for PC in HOL) which together explained the highest percentage of genetic variance of any trait (up to 37% for FC in HOL). Because significant effects are likely to be overestimated, it is possible that the percentage of variance explained by each QTL may have been artificially high. The number of detected QTL was rather limited. This is explained by the very conservative detection threshold used (P ≤ 6.10^−9^; − log_10_(P) ≥ 8.2) that decreased power of detection and excluded the QTL with smaller effects. For example, by decreasing the detection threshold to − log_10_(P) = 7 (P ≤ 6.10^−7^), we identified two additional QTL for CM in MON and HOL. These were located in a single genomic region around 88.5 Mb on BTA6 in the region of the *GC* gene, where we had found significant effects on udder morphology traits and SCS (Fig. 5).

#### QTL confirmation rate

In spite of the application of a very strict detection threshold to the bull GWAS results, about one-third of these QTL were not found in the cow populations. Several explanations could explain this situation. First, it is important to note that nearly all the highly significant QTL and all the QTL present in several breeds or affecting several traits were confirmed. A few QTL with − log_10_(P) > 10 were not confirmed but this was due to technical problems, the best selected variants being lost during the design of the chip. Most unconfirmed QTL, especially for udder conformation (10 out of 16), were detected in the bull population with − log_10_(P) values between 8.2 and 10 and had − log_10_(P) < 6 in the cow populations. Two reasons may be advocated. For these QTL, annotations were frequently very poor and we may have selected inappropriate variants. This point is especially critical when a small number of variants was selected. Indeed, due to our selection strategy, the enrichment in candidate variants increased with significance level in the bull populations, QTL size, and QTL sharing across breeds and traits, and the smallest QTL received a small number of candidate variants. In addition, we cannot exclude that some results that were unconfirmed in the cow population represent false positives.

#### QTL shared among breeds and traits

Although our results may have been shaped by factors specific to the breeds, traits, and populations analyzed here, still we successfully identified and validated QTL which were shared among more than one breed or related trait.

#### QTL shared among breeds

Five QTL associated with milk production traits were shared among all three breeds, while six other QTL were found in two of the three breeds (2 QTL for production and 4 for udder traits). Most of the QTL found in two breeds were shared between HOL and MON (4 QTL), probably because these were the two breeds FOR which we found the largest number of QTL. As mentioned above, the very strict detection threshold applied for the bull GWAS excluded some potential variants that also mapped at the same location for the same trait in another breed; thus, this reduced the number of significant results shared between breeds. For example, in the CI of QTL ID 42 (BTA5), detected for UD in HOL, we found a variant at 88,862,824 bp that also had, for the same trait, a significant effect in MON cows (− log_10_(P) = 7.4, results not shown) and an effect close to significance in MON bulls (− log_10_(P) = 7.2).

With these results, we were able to validate QTL shared among breeds for certain traits of interest. However, as previously reported from other studies conducted in multiple breeds at the nucleotide-level resolution [19,23], the variants with the most significant effects for a given trait differed largely among breeds. The reason for this result remains unclear. This could indicate that the causal mutations differed across breeds, but it may also be the result of differences in the quality of imputation of candidate variants among breeds. Within a QTL region, the effects of variants with the highest imputation accuracy, which are not necessarily the same across breeds, were probably estimated more accurately and were thus more likely to be significant. As shown later in Discussion, this hypothesis seems to be supported by the fact that, for several QTL detected in more than one breed, a shared variant often ranked highly among the best significant variants, even if it was not the very best. Precise identification of causal variants is further complicated by the presence of strong LD over large regions beyond the gene level.

#### QTL shared among traits

Within a breed, many of the QTL that we detected had effects on more than one production or udder trait (morphology and health). For example, in HOL, the QTL linked with production traits on BTA20 had effects on MY, PC, and FC, whereas the QTL identified on BTA29 for udder traits affected UD, UB, and SCS. These results are consistent with estimates of genetic correlations between milk yield and milk composition traits [24] and between udder health and udder morphology traits [7,8]. However, although significant genetic correlations were reported between milk production and udder morphology or udder health traits [8], we were not able to identify any QTL that had overlapping CI for production and udder (morphology or health) traits, even when we considered those with less-significant effects for CM (P ≤ 6·10^−7^). As an example, the QTL found on BTA6, which had effects on both udder health and morphology in all three breeds (Fig. 5), was located in the vicinity of another QTL that was detected in all three breeds and had effects on PC or PY. Depending on the breed, the variants with the most significant effects on udder traits were located between 88.5 and 88.9 Mb, while those with the most significant effects on production traits were located between 87 and 87.6 Mb. This region of the bovine genome (86–90 Mb) has been the subject of particular interest over the last ten years for its effects on milk production and udder health (clinical mastitis and somatic cell scores) [25–28]; the two most recent studies, both performed at the nucleotide level, identified a single variant or distinct but very close variants with the most significant effects on both milk yields and mastitis resistance [26,28]. Our study did not confirm the existence of a QTL with pleiotropic effects in this region; instead, our data suggest the presence of two neighboring QTL.

### Further investigations of QTL regions reveal the best candidate genes and variants

For QTL that were shared between breeds, and that had effects on multiple traits or were identified in both bulls and cows, the results obtained at the nucleotide level appeared to be very sensitive to the accuracies of phenotypes and genotypes. In most cases, the variant with the most significant effects differed among traits, among breeds or among populations within a breed (bulls vs. cows). However, in most of these QTL regions, a detailed investigation of the GWAS results revealed the genes and the variants that are most likely to be causative.

#### Candidate genes and variants for udder traits

The QTL that were confirmed to have the most significant effects on udder traits were located on BTA5, 6, and 14. In these three regions, GWAS results pinpointed *ABCC9*, *GC*, and *PLAG1* as the candidate genes, respectively.

The *ABCC9* (*ATP binding cassette subfamily C member 9*) gene, located on BTA5, was associated here with UD and FUA in HOL bulls and cows. In both analyses, the variant(s) with the most significant effects for both traits were located in intronic regions of the *ABCC9* gene, but approximately 12 to 24 kb apart: at 88,800,994 bp (rs110461240) in bulls and at 88,812,245 bp and 88,824,857 bp (rs209585944 and rs209893772, respectively, with the same significance level) in cows. This gene has previously been linked with milk production and fertility [23] and more recently with udder morphology (UD and FUA), milk production (MY and PY), and daughter pregnancy rate [29] in Holstein cattle. However, Jiang et al. [29], who performed a multi-trait analysis at the sequence level, failed to detect shared variants associated with different trait groups, suggesting the existence of several causal mutations for the different traits. In their study, the variants with the most significant effects were located at 88,818,703 bp (intron) for FUA and at 88,823,164 bp (splice region) for UD, i.e. between the most plausible candidate variants that we identified here. In our study, the best candidate variants identified by Jiang et al. [29] were confirmed to have very significant effects on UD (P = 4.3·10^−13^ and 4.4·10^−13^, respectively) and FUA (P = 1.3·10^−11^ and 1.4·10^−11^, respectively). In a nearby region, we also found significant effects on PY in HOL bulls but we could not confirm this in cows; the most significant variant in that case was the same as the one we detected for udder traits (rs136903701; 88,830,128 bp) but did not reach the level of significance (− log_10_(P) = 5.5). The *ABCC9* gene encodes a protein involved in the formation of the ATP-sensitive potassium channels in different muscles. These channels are expressed in many tissues and regulate different cellular functions; thus, mutations in the *ABCC9* gene could have potential effects on many traits.

As mentioned earlier, the region around 88.7 Mb on BTA6 has previously been linked with mastitis resistance [28,30,31] or udder morphology [32]. Here, we detected effects of this region on both type of traits—SCS in HOL and UD in HOL and MON—and, furthermore, it was the only region associated with mastitis resistance that we successfully validated in cows. Interestingly, the candidate variant that had the most significant effects (rs436532576; 88,723,742 bp) on these two traits in these two breeds in the validation GWAS was the most plausible causative variant previously identified in Red Danish [30,31] and German Fleckvieh cattle [32]. The effect of this candidate variant did not reach the level of significance in NOR (P = 4.10^−3^), but in NOR, the MAF of this variant was lower (0.21) than in MON (0.37) and HOL (0.40). The rs436532576 variant is located in an intronic region of *GC* (*vitamin D binding protein*), which was previously proposed as a candidate gene for resistance to mastitis in cattle because it encodes a Gc-globulin that is involved in both the transport of vitamin D to monocytes and phagocytic activity in macrophages [31].

On BTA14, the most plausible causative variants were identified in the *PLAG1* (*PLAG1 zinc finger*) gene in both MON bulls and cows. In the GWAS performed on imputed WGS of bulls, the variant with the most significant effects on RUH was located in the 5′-UTR region of *PLAG1* (rs210030313). Unfortunately, for technical reasons, it was not possible to add this variant to the customized chip. Instead, the variant with the most significant effects on this trait in MON cows was an intronic variant in *PLAG1*, located at 25,015,640 bp on BTA14 (rs109815800). The *PLAG1* gene has been associated with stature in cattle [1,33] and humans [34] but also with udder morphology [32]; variant rs109815800, which is a SNP on the Illumina Bovine HD BeadChip, was the most strongly associated of the whole-genome sequence variants with stature in the bovine meta-analysis of Bouwman et al. [1] and with udder depth in the study of Pausch et al. [32]. However, in our study, this variant was ranked 3rd by the sequence-based GWAS, after the 5′-UTR variants located at 25,052,440 bp (1st) and 25,052,394 bp (2nd). These two variants are also plausible causal variants as they present a higher probability of being located within a transcription binding site. Moreover, Pausch et al. [32], who found no association when UD was conditioned on body height, suggested that the association between *PLAG1* and udder morphology traits could be the result of phenotypic variation in body size rather than a true effect on mammary gland morphology. In our study, the lack of a significant effect of PLAG1 on other udder morphology traits than UD (less dependent on stature than UD) tends to support this hypothesis.

We identified and confirmed the effects on mammary gland morphology of other candidate variants on BTA5 in an intron of *TMTC2* (*transmembrane O*-*mannosyltransferase targeting cadherins 2*), on BTA20 upstream of *ISL1* (*ISL LIM Homeobox 1*), and on BTA26 in the 3′-UTR of *RAB11FIP2* (*RAB11 family interacting protein 2*). *TMTC2* was previously found to be associated with six udder type traits by Jiang et al. [29]. Instead, no such relationship has been reported for either *ISL1*, which encodes a member of the LIM/homeodomain family of transcription factors, or *RAB11FIP2*.

#### Candidate genes and variants for production traits

Among the genes that we identified here as being associated with milk production and composition traits, there are a number of well-characterized functional candidate genes: *GHR*, which encodes a growth hormone receptor, *PAEP* and *CSN2*, which encode milk proteins, and *DGAT1*, *GPAT4* and *FASN*, all of which encode enzymes involved in the metabolism of fatty acids in milk. We also identified several other candidate genes with less well known functions or for which functional links with dairy traits have not yet been established: *MGST1*, *CDDC57*, *TBC1D22A*, *VPS13B*, *PICALM*, and *GRAMD4*.

The F279Y missense mutation in the *GHR* (*growth hormone receptor*) gene, which has previously been implicated in the genetic variation of PC and FC [35,36], had the most significant effects on PC (P = 1.2·10^−102^) and FC (P = 4.4·10^−55^) in HOL cows, and was ranked 2nd for MY (P = 1.4·10^−10^), confirming the QTL region identified in HOL bulls. The allele responsible for a decrease in the protein and fat contents of milk had a frequency of 0.12. In the GWAS performed on bulls, the *F279Y* variant had very significant effects on PC (P = 7.9·10^−23^) but the variant with the most significant effects in this region was an intergenic variant located at 32,254,539 bp (P = 2.5·10^−30^), i.e. relatively distant (~ 250 kb) from the causal mutation; this suggested poor imputation accuracy in the region surrounding *GHR*. No effects of this region were detected in NOR and MON cows, but the MAF of the *F279Y* variant was much lower in these two breeds (0.07 and 0.006, respectively). In contrast to Viitala et al. [35], we found no significant effects of the *S18N* variant in the *PRLR* (*prolactin receptor*) gene, located approximately 7 Mb downstream of *GHR*, on any of the milk production traits, although this missense mutation was polymorphic in MON, NOR, and HOL (MAF = 0.23, 0.42 and 0.16, respectively). Our result corroborates the hypothesis that the *S18N* mutation in *PRLR* may not be causative but is instead, at least in populations in which its effects have been demonstrated, in LD with the causal mutation [37].

In HOL, we identified and validated two QTL located near the *PAEP* (*progestagen associated endometrial protein*) gene, which encodes β-lactoglobulin (BTA11 at ~ 103.3 Mbp), and likewise confirmed the effects of the cluster of casein genes encoding the αs1 (*CSNS1*), αs2 (*CSNS2*), β (*CSN2*), and κ (*CSN3*) caseins (BTA6 at ~ 87.2 Mbp) in MON, NOR, and HOL. Although our results differed depending on the breed and population (bulls or cows) analyzed, *PAEP* and *CSN2* were found to be the best candidate genes in HOL cows for the QTL acting on FC and PY, respectively. The best candidate variant in *CSN2* was the missense variant responsible for the A1/B and A2 protein variants (at 87,181,619 bp; rs43703011), which has previously been implicated in milk composition and cheese-making quality [38]. This variant also had very significant effects on PC in all three breeds (MON P = 8.8·10^−28^, MAF = 0.38; NOR P = 7.2·10^−13^, MAF = 0.28; and HOL P = 9.8·10^−11^, MAF = 0.33) but it was not ranked among the top 10 variants of the peak for this trait. We also detected and confirmed another QTL on BTA6 in HOL in the region of the *ABCG2* gene previously identified for milk composition [39]. Only two of the 138 variants with significant effects on FC and/or PC in Holstein bulls, located in the EXT-CI of the QTL (37.5–38.5 Mb), were in the *ABCG2* gene (rs136230937 at 38,015,146 bp and rs110063427 at 38,020,110 bp). They are intronic and therefore distinct from the rs43702337 missense variant (at 38,027,010 bp) described by Cohen-Zinder et al. [39]. Moreover, both variants were much less significant (–log_10_(P) = 7.4 for FC and 16.8 for PC) than the variant with the most significant effect on both traits, located in the *HERC6* gene (intron) (– log_10_(P) = 9.7 for FC and 24.3 for PC). Thus, in our study *ABCG2* is not the best candidate gene. However, we cannot completely exclude it because of its low MAF (0.02) and therefore its limited imputation accuracy, which may tend to underestimate its effect. For the QTL on BTA11 that affected FC in HOL cows, the 10 most significant variants were all located in the *PAEP* gene. Six of them were identified in a 1.5-kb stretch of the upstream region of the gene (103,299,655–103,301,229 bp), and were ranked from 1st (103,300,548 bp; rs109982707) to 8th in the peak; the 4th-ranked variant was in the 5′-UTR region (103,301,694 bp; rs41255686); the 6th-ranked variant was located in the downstream region (103,308,330 bp; rs109087963); the 9th-ranked variant was in a splicing region (103,304,656 bp; rs109990218); and finally, the 10th-ranked variant in the peak was a missense variant (103,303,475 bp; rs110066229). Together with another missense variant located at 103,304,757 bp (rs109625649), variant rs110066229 was previously identified as the functional mutation for protein variants A and B, which are associated with different levels of β-lactoglobulin in milk [40]. Several nucleotide-level GWAS have found effects of this region on FC [23,29,41] or milk whey proteins [19,42], and all have pointed to candidate variants in the *PAEP* gene. However, each of these studies highlighted a different best candidate variant, and these variants were always distinct from the two missense variants that cause the A and B protein polymorphisms. Moreover, Sanchez et al. [19] found that a peak remained when one of the missense variants was fixed in the GWAS, which suggested that the missense variants described by Ganai et al. [40] do not explain all the effects of this region on milk composition.

We also identified several genes involved in the metabolism of milk fatty acids (*FASN*, *DGAT1*, *GPAT4*, and *MGST1*) as good functional candidates to explain the changes observed in milk composition, and in each of these genes, we highlighted the most plausible candidate variants. *FASN* (*fatty acid synthase*) encodes a key enzyme in de novo fatty acid synthesis, whereas *GPAT4* (*glycerol*-*3*-*phosphate acyltransferase 4*) is paralogous to *DGAT1* (*diacylglycerol O*-*acyltransferase 1*), with the two genes occupying adjacent nodes of the mammary triglyceride synthesis chain [43]. The *MGST1* (*microsomal glutathione S*-*transferase 1*) gene plays a role in oxidative stress reaction and although it has typically been associated with milk composition, and in particular with milk fat, its role in lipid metabolism is less clear. It has been shown to reduce lipid peroxidation products in human mammary cell culture [44], but its functional impact on bovine milk production or composition traits has not been yet demonstrated.

The QTL region that was detected and validated at the centromeric end of BTA14 presented effects on different milk production traits, with the strongest effect on FC in the three breeds. With a frequency of 0.22, the *A* allele of the *K232A* mutation in *DGAT1*, which decreases FC, PC, and FY, and increases MY and PY [45], was the most significant variant for FC in HOL cows. It ranked 3rd and 13th in the peak for this trait and was much less polymorphic in NOR (MAF = 0.08) and MON (MAF = 0.007), respectively. In the vicinity of *DGAT1*, many genes have been annotated in the 0.5-Mb region between 1.5 and 2 Mb on BTA14. Our analyses indicated that the best candidate variants for many other traits in different breeds were located in other genes of this region (*MROH1*, *bta*-*mir*-*1839*, *HSF1*, *RECQL4*, *MFSD3*, *GPT*, *CPSF1*, *ADCK5*, and *SLC39A4*); further investigations could reveal, as has been suggested in many dairy cattle breeds and in particular in HOL, MON, and NOR [46], the existence of other causal mutations in this region.

We also identified two other candidate genes acting on FC in the QTL detected on BTA19 in HOL bulls and cows. In HOL cows, the variants with the most significant effects were both missense and located in the *CCDC57* gene (rs41921161 at 51,319,797 bp, ranked 1st, and rs41921160 at 51,319,759 bp, ranked 2nd). However, five variants in the *FASN* gene ranked 5th to 9th in the peak with three located in the upstream region and two intronic. Among these, the upstream rs136067046 variant (at 51,383,847 bp, ranked 6th) was also the best candidate variant identified in a previous study for a QTL acting on milk fatty acid composition [42]. This region has been extensively studied for its effects on milk fat content and milk fatty acid composition. Although the role of *FASN* in the regulation of milk fat is more obvious than that of *CCDC57*, both genes are generally cited to explain the effects of this region [47–50].

In the three breeds studied here, we found a QTL on BTA27 that was also strongly associated with FC. The results of the cow GWAS directly pointed to five candidate variants, all located in the *GPAT4* gene, which ranked in the top 5 in all three breeds. These variants, which were in complete LD in each of the three breeds, had a MAF ranging from 0.47 to 0.49 depending on the breed and are located in the upstream (rs211250281, rs378026790, rs209479876, and rs209855549) or the 5′-UTR (rs208675276) regions of *GPAT4*. *GPAT4*, also named *AGPAT6*, was previously described as a functional gene for milk fat content as well as protein and lactose contents by Littlejohn et al. [51]. These authors identified 10 linked variants associated with milk composition, which included the rs211250281, rs209855549, and rs208675276 variants found in the top 5 for each of the three breeds analyzed in our study. Moreover, the four variants located in the upstream region have been identified as candidate causal variants for FC in Holstein and Fleckvieh cows [2] whereas the top five variants were found to be the best candidates to explain variations in milk protein composition in a multi-breed analysis [19] and variations in fat content in a meta-analysis [23]. All these results are consistent with the existence of a causative mutation located in the promoter region of *GPAT4* which could regulate the expression level of this gene. Daetwyler et al. [2] suggested that the InDel rs378026790 was the most likely causal variant because of its high probability to overlap a transcription factor binding site but we cannot exclude rs208675276 which is in the 5′-UTR region and therefore closer to the transcription initiation site.

*MGST1* has also been frequently described as a functional candidate gene for the QTL detected at ~ 94 Mb on BTA5 with effects on milk composition traits [19,23,41,42,52–54]. In the present study, the variant, which was shared between MON, NOR, and HOL cows and was most strongly associated with fat content or yield, was located in an intronic region of this gene (rs211210569 at 93,945,738 bp). This variant was also found to be responsible for effects on fat yield in the study of van den Berg et al. [52] in both Danish and French Holstein bulls.

In addition to these good functional genes, we also identified and validated other promising genes for which the relationship with milk production or composition traits is less thoroughly understood. *PICALM* (*phosphatidylinositol binding clathrin assembly protein*), which was linked with PC in HOL (on BTA29), was previously associated with milk protein composition and lactose content [19,42,55]. *TBC1D22A* (*TBC1 domain family member 22A*) was associated with PC in HOL and has been previously implicated in milk protein content [23,29]. *VPS13B* (*vacuolar protein sorting 13 homolog B*) had effects on FC, PC, and MY in our study and has been previously associated with milk fat and protein contents [56]. Finally, *GRAMD4* (*GRAM domain containing 4*) had effects on PC in MON and was previously identified as a candidate gene for milk protein and mineral composition in the same breed [42].

The candidate variants that we identified in this study for both production and udder traits, which were mostly located in the non-coding regions of the genome, are either causative themselves or in LD with causative variants. The discovery of causal variants for complex traits remains challenging but should be facilitated in the next few years by two factors: (i) the most recent run of the 1000 Bull Genomes Project (run8 released in 2020), which contains, in total and within each breed, a larger number of bovine animals with whole-genome sequences that are aligned on the most recent ARS-UCD1.2 bovine genome assembly [57] to enable more accurate imputation, and (ii) improved annotations of regulatory regions of the bovine genome, provided by the FAANG consortium [58].

## Conclusions

In the current study, GWAS analyses conducted on 10,871 bulls and 85,303 cows of the three main French dairy cattle breeds, Holstein, Montbéliarde, and Normande, enabled the identification and validation of 54 QTL for economically important traits related to milk production, udder morphology, and udder health. The first set of GWAS was carried out using whole-genome sequence data from bulls for the purpose of primary detection, and these enabled us to directly target candidate genes and candidate variants that were then added to the customized chip used for routine genomic evaluation of French dairy cattle. Analyses conducted in younger populations of cows then enabled us to validate a large number of these genes and variants, and yielded a more comprehensive understanding of the genetic determinism underlying these traits. Because they are now included on the genotyping chip, these candidate causative variants can be used for genomic predictions of production and udder traits in these three dairy cattle breeds.

## Supplementary information

**Additional file 1: Table S1.** Heritability of traits in Montbéliarde (MON), Normande (NOR), and Holstein (HOL) cattle.

## Data Availability

Phenotypes originated from the national database for genetic evaluation. Most of the cow genotypes originated from genomic selection programs that are managed by Valogene. All data belong to French farmers and cannot be disclosed without explicit authorization.
