# Supplementary material for: Confirmed effects of candidate variants for milk production, udder health, and udder morphology in dairy cattle
Source: Genet Sel Evol. 2020 Oct 1;52:55. doi: 10.1186/s12711-020-00575-1 (PMC7529513; doi:10.1186/s12711-020-00575-1)
Supplement: Supplementary file 1 — Additional file 1: Table S1. Heritability of traits in Montbéliarde (MON), Normande (NOR), and Holstein (HOL) cattle. [file 12711_2020_575_MOESM1_ESM.docx]

**Table S1.** Heritability of traits in Montbéliarde (MON), Normande (NOR), and Holstein (HOL) cattle

| Type of trait | Trait and abbreviation | Heritability of trait | | |
| --- | --- | --- | --- | --- |
|  |  | MON | NOR | HOL |
| Milk production | Milk Yield (kg) MY | 0.30 | 0.30 | 0.30 |
|  | Fat Content (%) FC | 0.50 | 0.50 | 0.50 |
|  | Protein Content (%) PC | 0.50 | 0.50 | 0.50 |
|  | Fat Yield (kg) FY | 0.30 | 0.30 | 0.30 |
|  | Protein Yield (kg) PY | 0.30 | 0.30 | 0.30 |
| Udder health | Clinical Mastitis CM | 0.023 | 0.021 | 0.018 |
|  | Somatic Cell Score SCS | 0.15 | 0.15 | 0.15 |
| Udder morphology | Udder Support US | 0.22 | 0.39 | 0.23 |
|  | Udder Depth UD | 0.38 | 0.31 | 0.36 |
|  | Fore Udder Attachment FUA | 0.27 | 0.28 | 0.25 |
|  | Rear Udder Height RUH | 0.25 | 0.18 | 0.21 |
|  | Teat Length TL | 0.45 | - | 0.39 |
|  | Fore Teat Distance FTD | 0.34 | 0.38 | 0.35 |
|  | Udder Balance UB | 0.15 | 0.27 | 0.22 |
|  | Teat Orientation TO | 0.28 | 0.35 | 0.29 |
| Milking ease | Milking Speed Score MSS | 0.27 | 0.25 | 0.20 |
